# AutoBend: An Automated Approach for Estimating Intervertebral Joint Function from Bone-Only Digital Models

**DOI:** 10.1093/iob/obab026

**Published:** 2021-10-13

**Authors:** K E Jones, R J Brocklehurst, S E Pierce

**Affiliations:** Museum of Comparative Zoology and Department of Organismic and Evolutionary Biology, Harvard University, 26 Oxford Street, Cambridge, MA 02138, USA; Department of Earth and Environmental Sciences, University of Manchester, Williamson Building, Oxford Road, Manchester M13 9PL, UK; Museum of Comparative Zoology and Department of Organismic and Evolutionary Biology, Harvard University, 26 Oxford Street, Cambridge, MA 02138, USA; Museum of Comparative Zoology and Department of Organismic and Evolutionary Biology, Harvard University, 26 Oxford Street, Cambridge, MA 02138, USA

## Abstract

Deciphering the biological function of rare or extinct species is key to understanding evolutionary patterns across the tree of life. While soft tissues are vital determinants of joint function, they are rarely available for study. Therefore, extracting functional signals from skeletons, which are more widely available via museum collections, has become a priority for the field of comparative biomechanics. While most work has focused on the limb skeleton, the axial skeleton plays a critical role in body support, respiration, and locomotion, and is therefore of central importance for understanding broad-scale functional evolution. Here, we describe and experimentally validate AutoBend, an automated approach to estimating intervertebral joint function from bony vertebral columns. AutoBend calculates osteological range of motion (oROM) by automatically manipulating digitally articulated vertebrae while incorporating multiple constraints on motion, including both bony intersection and the role of soft tissues by restricting excessive strain in both centrum and zygapophyseal articulations. Using AutoBend and biomechanical data from cadaveric experiments on cats and tegus, we validate important modeling parameters required for oROM estimation, including the degree of zygapophyseal disarticulation, and the location of the center of rotation. Based on our validation, we apply a model with the center of rotation located within the vertebral disk, no joint translation, around 50% strain permitted in both zygapophyses and disks, and a small amount of vertebral intersection permitted. Our approach successfully reconstructs magnitudes and craniocaudal patterns of motion obtained from *ex vivo* experiments, supporting its potential utility. It also performs better than more typical methods that rely solely on bony intersection, emphasizing the importance of accounting for soft tissues. We estimated the sensitivity of the analyses to vertebral model construction by varying joint spacing, degree of overlap, and the impact of landmark placement. The effect of these factors was small relative to biological variation craniocaudally and between bending directions. We also present a new approach for estimating joint stiffness directly from oROM and morphometric measurements that can successfully reconstruct the craniocaudal patterns, but not magnitudes, derived from experimental data. Together, this work represents a significant step forward for understanding vertebral function in difficult-to-study (e.g., rare or extinct) species, paving the way for a broader understanding of patterns of functional evolution in the axial skeleton.

## Abstract (pt)

**Resumo [Portuguese]** Decifrar a função biológica de espécies raras ou extintas é fundamental para se compreender os padrões evolutivos na árvore da vida. Embora os tecidos moles sejam determinantes vitais das funções articulares, estes raramente estão disponíveis para estudo. Portanto, extrair dados funcionais provenientes de esqueletos, que são mais amplamente disponíveis por meio de coleções de museus, tornou-se uma prioridade para o campo da biomecânica comparada. Embora a maioria dos trabalhos biomecânicos tenham focado no esqueleto apendicular, o esqueleto axial também desempenha um papel crítico para o suporte corporal, respiração e locomoção e, portanto, é de importância central para a compreensão da evolução funcional em escalas amplas. Nesse trabalho, nós descrevemos e validamos experimentalmente o AutoBend, uma abordagem automatizada para estimar a função da articulação intervertebral de colunas vertebrais ósseas. O AutoBend calcula a amplitude do movimento osteológico (AMO) manipulando automaticamente as vértebras reconstruídas digitalmente e incorporando várias restrições de movimento, incluindo restrições das interseções ósseas e o papel de restrição dos tecidos moles na tensão excessiva sobre as articulações dos centros vertebrais e zigapofisárias. Usando AutoBend e dados biomecânicos de experimentos cadavéricos em gatos e lagartos tegus, validamos parâmetros de modelagem importantes e necessários para as estimativa do AMO, incluindo o grau de desarticulação zigapofisária e a localização do centro de rotação. Com base nessa validação, aplicamos um modelo com o centro de rotação localizado dentro do disco vertebral, sem translação articular, com cerca de 50% de tensão permitida nas zigapófises e discos vertebrais, além de uma pequena quantidade de intersecção vertebral permitida. Nossa abordagem reconstrói com sucesso magnitudes e padrões de movimento craniocaudais obtidos a partir de experimentos *ex vivo*, corroborando a sua potencial utilidade. Esse modelo também tem um desempenho melhor do que os métodos mais típicos que dependem apenas das interseções ósseas, enfatizando a importância de se levar em conta o papel dos tecidos moles. Estimamos a sensibilidade das análises à reconstrução do modelo vertebral, variando o espaçamento entre articulações, o grau de sobreposição e o impacto da localização dos pontos de referência. O efeito desses fatores foi pequeno em relação à variação biológica craniocaudal e entre as direções de flexão. Apresentamos aqui também uma nova abordagem para se estimar a rigidez articular diretamente à partir do AMO e medidas morfométricas que podem reconstruir com sucesso os padrões craniocaudais (embora não as magnitudes) derivados de dados experimentais. Este trabalho representa um passo significativo para a melhor compreensão da função vertebral em espécies difíceis de estudar (por exemplo, raras ou extintas), abrindo caminho para uma compreensão mais ampla dos padrões de evolução funcional no esqueleto axial.

## Abstract (es)

**Resumen [Spanish]** Descifrar la función biológica de especies raras o extintas es fundamental para comprender los patrones evolutivos del árbol de la vida. Aunque los tejidos blandos son determinantes vitales de la función articular, raramente están disponibles para su estudio. Por lo tanto, la extracción de datos funcionales de esqueletos, que están más comumente disponibles a través de colecciones de museos, se ha convertido en una prioridad para el campo de la biomecánica comparada. Aunque la mayor parte del trabajo biomecánico se ha centrado en el esqueleto apendicular, el esqueleto axial también desempeña un papel fundamental para el soporte del cuerpo, la respiración y la locomoción y, por lo tanto, es de vital importancia para comprender la evolución funcional a gran escala. En este trabajo, describimos y validamos experimentalmente AutoBend, una herramienta automatizada para estimar la función de la articulación intervertebral en las columnas vertebrales óseas. AutoBend calcula el rango de movimiento osteológico (RMOo) manipulando automáticamente las vértebras reconstruidas digitalmente e incorporando varias restricciones de movimiento, incluidas las restricciones de intersección ósea y el papel de la restricción de tejidos blandos en la tensión excesiva sobre las articulaciones cigapofisarias y centros vertebrales. Utilizando AutoBend y datos biomecánicos de experimentos cadavéricos en gatos y lagartos tegus, validamos importantes parámetros de modelado necesarios para estimar el RMOo, incluido el grado de desarticulación cigapofisaria y la ubicación del centro de rotación. Con base en esta validación, aplicamos un modelo con el centro de rotación ubicado dentro del disco vertebral, sin traslación articular, con 50% de tensión permisible en la cigapófisis y los discos vertebrales, además de una pequeña cantidad de intersección vertebral permitida. Nuestra herramienta reconstruye con éxito magnitudes craneocaudales y patrones de movimiento obtenidos de experimentos *ex vivo*, corroborando su potencial utilidad. Este modelo también funciona mejor que los métodos más típicos que se basan solo en las intersecciones óseas, enfatizando la importancia de tener en cuenta el papel de los tejidos blandos. Estimamos la sensibilidad de los análisis a la reconstrucción del modelo vertebral, variando el espaciamiento entre articulaciones, el grado de superposición y el impacto de la ubicación de los puntos de referencia. El efecto de estos factores fue pequeño en relación a la variación biológica craneocaudal y entre las direcciones de flexión. También presentamos aquí un nuevo enfoque para estimar la rigidez articular directamente de la RMOo y mediciones morfométricas que pueden reconstruir con éxito los patrones craneocaudales (aunque no las magnitudes) derivados de datos experimentales. Este trabajo representa un paso significativo hacia una mejor comprensión de la función vertebral en especies difíciles de estudiar (por ejemplo, raras o extintas), allanando el camino para una comprensión más amplia de los patrones de evolución funcional en el esqueleto axial.

## Introduction

Most of our understanding of organismal function is based on select model taxa that are easily studied in a laboratory setting; however, this approach fails to account for the majority of living and extinct biodiversity (Lauder and Thomason 1995). The vast collections in natural history museums, combined with recent advances in scanning and digitization efforts, have placed an enormous wealth of osteological data at our fingertips (Boyer et al. 2016; Nelson and Ellis 2019; Hedrick et al. 2020). These data represent an untapped resource for studying organismal function if techniques can be developed to exploit them (Muñoz and Price 2019). Therefore, establishing the relationship between bony morphology and function is of central importance as it has the potential to provide more rigorous interpretations of broad-scale functional evolution (Molnar et al. 2021; Jones et al. 2021; Lungmus and Angielczyk 2019; Dickson et al. 2021).

One approach for estimating function from skeletons involves three-dimensional computer modeling, where biomechanical principals are applied to bones to empirically estimate function (Hutchinson and Garcia 2002; Nyakatura et al. 2019; Sellers et al. 2009). Modeling is particularly advantageous when the morphology of an organism falls outside that observed in available study species, making direct correlations impossible (Ibrahim et al. 2020; McHenry et al. 2007). For example, understanding how joint mobility varies within and between species can shed light on changes in musculoskeletal function (Pierce, Clack, and Hutchinson 2012; Manafzadeh, Kambic, and Gatesy 2021; Gatesy 1991; Kambic, Roberts, and Gatesy 2017; Lai, Biewener, and Pierce 2018; Molnar et al. 2021; Hutson and Hutson 2014, 2015). However, estimating mobility from skeletons is challenging. Validation of bone-only range of motion (aka osteological range of motion, oROM) against cadaveric studies in the appendicular skeleton reveals that joint mobility tends to be overestimated (Tsai et al. 2020; Kambic, Biewener, and Pierce 2017; Arnold, Fischer, and Nyakatura 2014; Hutson and Hutson 2012), highlighting the important role of soft tissues in determining joint function. Further, the relationship between ROM in cadavers and *in vivo* mobility during specific behaviors is complex, and has rarely been explored in detail. In lizards, alligators and birds, total hindlimb ROM was far lower during *in vivo* behaviors such as walking and running than during cadaveric manipulation, but the overall patterns of mobility remained consistent between *ex vivo* and *in vivo* contexts (Manafzadeh, Kambic, and Gatesy 2021; Arnold, Fischer, and Nyakatura 2014). Further, while little *in vivo* data exist for the vertebral column, *ex vivo* joint mobility patterns correlate with locomotor behavior across multiple clades (Molnar et al. 2015; Jones et al. 2020; Nyakatura et al. 2019; Long et al. 1997). Therefore, although oROM cannot provide a direct measure of *in vivo* function, it provides a useful tool for understanding broad-scale comparative patterns where experimental data are not available (e.g., extinct organisms).

Considerable work has focused on reconstructing range of motion of the limbs to test locomotor hypotheses in extinct groups (Pierce, Clack, and Hutchinson 2012; Molnar et al. 2021; Reilly and Elias 1998; Gatesy 1991; Willey et al. 2004; Lai, Biewener, and Pierce 2018; Hutson and Hutson 2014; Nyakatura et al. 2019), but far fewer studies have considered oROM in the axial skeleton. The vertebral column is better suited to digital oROM analysis than the limbs because each vertebral motion segment, consisting of two vertebrae and an intervertebral joint, fits together tightly via a centrum articulation and at least two zygapophyseal synovial joints (Koob and Long 2000; Wintrich et al. 2020). This helps to constrain digital reconstruction of vertebral motion segments and limits the total mobility of the joint relative to other anatomical regions (Molnar et al. 2015; Oliver et al. 2016; Jones et al. 2020). Further, the vertebral column plays an important role in diverse behaviors such as locomotion, breathing, and thermoregulation (Buchholtz 1998; Brainerd and Owerkowicz 2006; Cieri et al. 2020; Carrier 1987; Schilling and Hackert 2006; Schilling 2011). Therefore, understanding the functional implications of vertebral changes is key to understanding numerous evolutionary transitions. For example, recent work has shown that the evolution of mammals from their synapsid forerunners is associated with complex changes in axial function that transcend the simple “lateral-to-sagittal” bending paradigm often touted for this group (Jones et al. 2021).

When applied to the vertebral column, digital oROM has mostly been restricted to the cervical column, with a particular focus on neck posture and mobility in a range of dinosaurs (Mallison 2010a, 2010b; Taylor and Wedel 2013), as well as fossil turtles (Werneburg et al. 2015) and plesiosaurs (Nagesan, Henderson, and Anderson 2018). However, cervical oROM has only been validated on a select few extant species such as ostriches (Cobley, Rayfield, and Barrett 2013), turkeys (Kambic, Biewener, and Pierce 2017), and giraffes (Vidal et al. 2020). Dorsal vertebral column oROM has been even less studied, though notably Molnar et al. (2015) used digital modeling to reconstruct thoracolumbar mobility in fossil crocodilians. Regardless of this work, consensus has yet to be reached about the best approach for determining oROM or the impact of their underlying assumptions. Generally, oROM estimation relies on manual manipulation of vertebrae in digital space, and visual assessment of constraints on motions (e.g., when two vertebrae intersect) that are highly subjective and are not repeatable. Automation of such approaches offers the opportunity to increase the speed and repeatability of vertebral oROM, as well as the potential to parameterize the impact of various sources of uncertainty on the model.

Several key assumptions underlie the creation of a vertebral oROM model. First, the vertebral motion segments must be reconstructed as accurately as possible. For many species, the intervertebral joints themselves are tightly fitting and so their orientation can be reconstructed reasonably well, but determining the exact spacing between vertebrae can be more challenging (Taylor and Wedel 2013). Second, the constraints on motion during the digital manipulation must be defined. Bony intersection (also known as “bony stops”) is universally applied as a constraint on motion, but if and how to account for the crucial role of soft tissue constraints is controversial. For example, some studies use 50% disarticulation of the zygapophyseal joint as a motion constraint (Molnar et al. 2015; Stevens and Parrish 1999), while others argue greater strain is possible (Cobley, Rayfield, and Barrett 2013; Kambic, Biewener, and Pierce 2017). Further, impacts of the centrum soft tissues (e.g., intervertebral disk, annular ligaments) in limiting motion have been largely ignored (although see finite element approach by Wintrich et al. 2019). Therefore, a more systematic approach for applying and quantifying the impact of soft tissues on vertebral oROM is required to fine-tune digital models of vertebral motion.

Here, we describe AutoBend, a novel, automated approach to estimating intervertebral joint oROM via digital bending experiments. AutoBend is fast, repeatable, and parameterized, and so can directly measure the sensitivity of models to various input parameters. We validate the technique against experimental data collected on two extant species with divergent morphologies and locomotor modes: a lizard (Argentine black and white tegu, *Salvator merianae*) and a mammal (domestic cat, *Felis catus)* (Jones et al. 2020). While tegus emphasize lateral bending of the back, cat spines are capable of considerable mobility in lateral bending, sagittal bending, and axial rotation, ensuring that a wide range of axial function is represented (Jones et al. 2020). In addition to bony intersection, AutoBend constrains motion using 3D landmarks that estimate strain at the zygapophyseal and centrum joints to account for soft tissues during bending. By varying the constraints applied and underlying model parameters, we test the impact of various model assumptions discussed in the literature, and assess the sensitivity of the model to noise associated with model construction and parameter estimation. We also propose a new method for the estimation of intervertebral joint stiffness from skeletal material using oROM and bony morphology of the vertebrae. Our approach accurately estimates both pattern and magnitude of intervertebral oROM and patterns of joint stiffness in these two taxa, demonstrating the importance of going beyond bony stops to account for soft tissues when reconstructing joint function from bony morphology alone.

## Materials and methods

### Overview

**Fig. 1:**
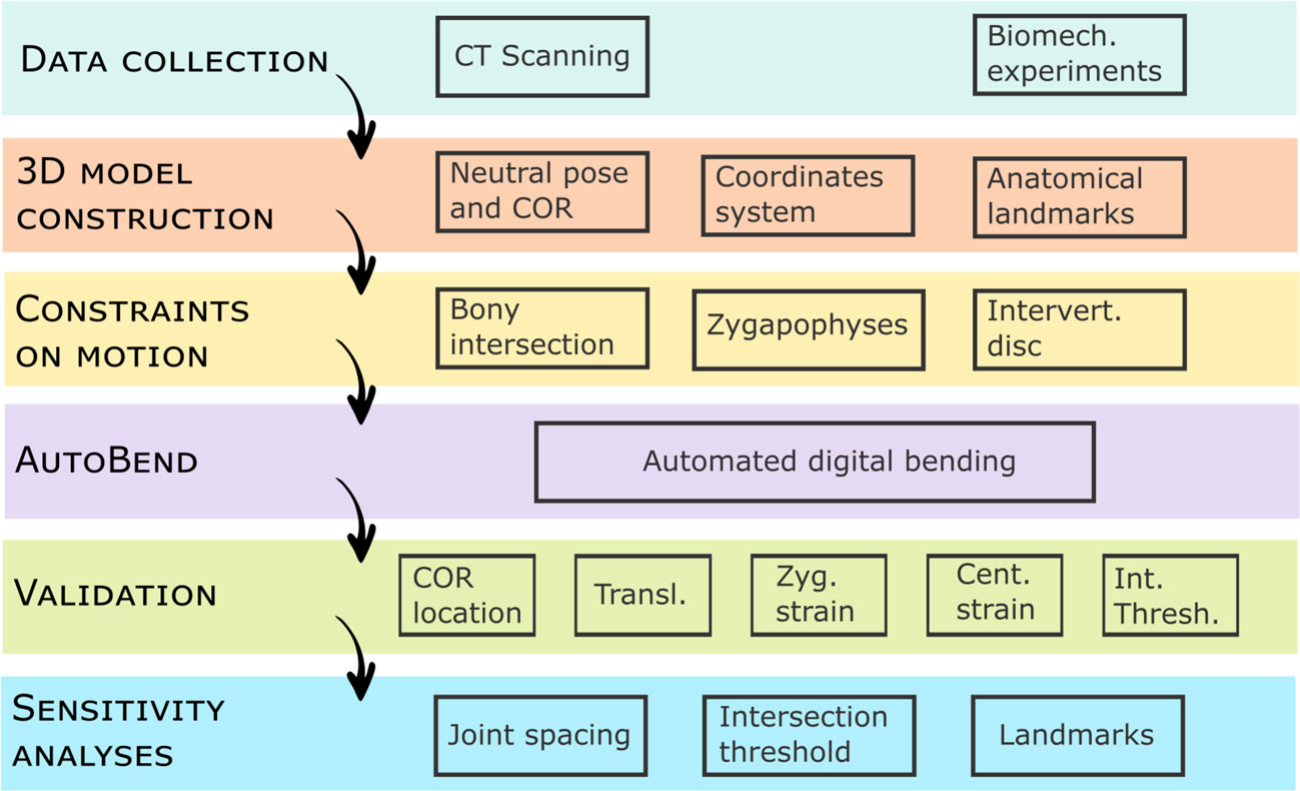
Flowchart of steps in the automated bending analysis.

We validate AutoBend against cadaveric experimental bending data from *F*. *catus* (domestic cat) and *S*. *merianae* (Argentine black and white tegu) (Jones et al. 2020) (Fig. 1). Vertebral columns were micro-computed tomography (CT) scanned and 3D digital models constructed of all presacral vertebral motion segments. The neutral pose, center of rotation (COR), and coordinate system were established, and then 10 anatomical landmarks were placed on each joint to estimate the impact of soft tissues during bending. Automated digital bending experiments (AutoBend) were run in Autodesk Maya software using custom Python code (freely available on github). AutoBend allows constraints on motion to be applied based on bony intersection, but it can also account for soft tissue displacement at the zygapophyses and intervertebral disk. First, analyses were conducted to test the validity of commonly applied oROM modeling parameters, and second to ascertain the sensitivity of the model to error associated with model construction and implementation. Validation tests were conducted on the location of the placement of the COR, the degree of translation permitted, the degree of zygapophyseal displacement permitted (zygapophysis strain), the degree of centrum displacement permitted (centrum strain), and the amount of bony intersection permitted. Once “best estimate” parameter combinations were selected, sensitivity analyses were run to determine confidence intervals on the estimation of mobility by repeating the bending experiments eight times for each joint, while varying joint spacing, intersection between vertebrae, and strain parameters to account for errors in mesh construction and landmark placement. Joint stiffness was estimated using oROM and vertebral morphology (as explained further later).

### Data collection

Biomechanical data were gathered from a previous study of 10 domestic cats and 8 Argentine black and white tegu lizards, in which intervertebral joint range of motion and compliance (inverse of stiffness) were measured using static bending experiments on cadaveric specimens (Jones et al. 2020). These experiments were conducted *ex vivo*, and thus represent the maximum possible intervertebral joint mobility and not mobility as utilized *in vivo* during a particular behavior. However, *ex vivo* joint mobility patterns have been shown to reflect large-scale variation in *in vivo* function across multiple clades (Molnar et al. 2015; Jones et al. 2020; Nyakatura et al. 2019; Long et al. 1997). Further, by directly comparing maximum mobility in cadavers and bone-only models we explore the impact of soft tissues on estimating vertebral oROM.

The presacral vertebrae from one domestic cat (SEP 38) and one Argentine black and white tegu lizard (SEP 103) were micro-CT scanned using a SkyScan 1173 micro-CT (Bruker Madison, WI, USA) as part of the original biomechanical study and segmented in Materialise (Plymouth, MI, USA) Mimics v.19 using the “split mask” tool (see more details in Jones et al. 2020). Individual vertebrae were imported into Materialise 3Matic v.14 as STL mesh format and the meshes were wrapped and smoothed to remove any uneven surfaces and fill any holes (e.g., from screws placed during biomechanical experiments).

### 3D model construction

Beginning with the first postaxial vertebra (C3), vertebrae were articulated posteriorly by fitting joints together using the N-points registration tool in 3Matic. Alignment points were selected in the center of each zygapophyseal facet and in the center of the centrum. The alignment was then tweaked using the interactive translate/rotate tool until the endplates were parallel and the zygapophyseal facets maximally overlapped. This was considered the “neutral pose” for the vertebral motion segment.

The COR was positioned in 3Matic using the create analytical sphere tool. Two different locations for the COR were applied, COR-disk and COR-zyg (see discussion later). For the COR-zyg, the sphere was created automatically using the four-point method, with two points on each post-zygapophyseal facet of the anterior vertebra at its cranial and caudal extreme. For the COR-disk in the cat, a sphere was placed at the center of the endplate of the anterior vertebra, then translated to the middle of the intervertebral space in lateral view. In the tegu, a sphere was fit to the surface of the proceolous ball joint of the anterior vertebra in the motion segment.

**Fig. 2:**
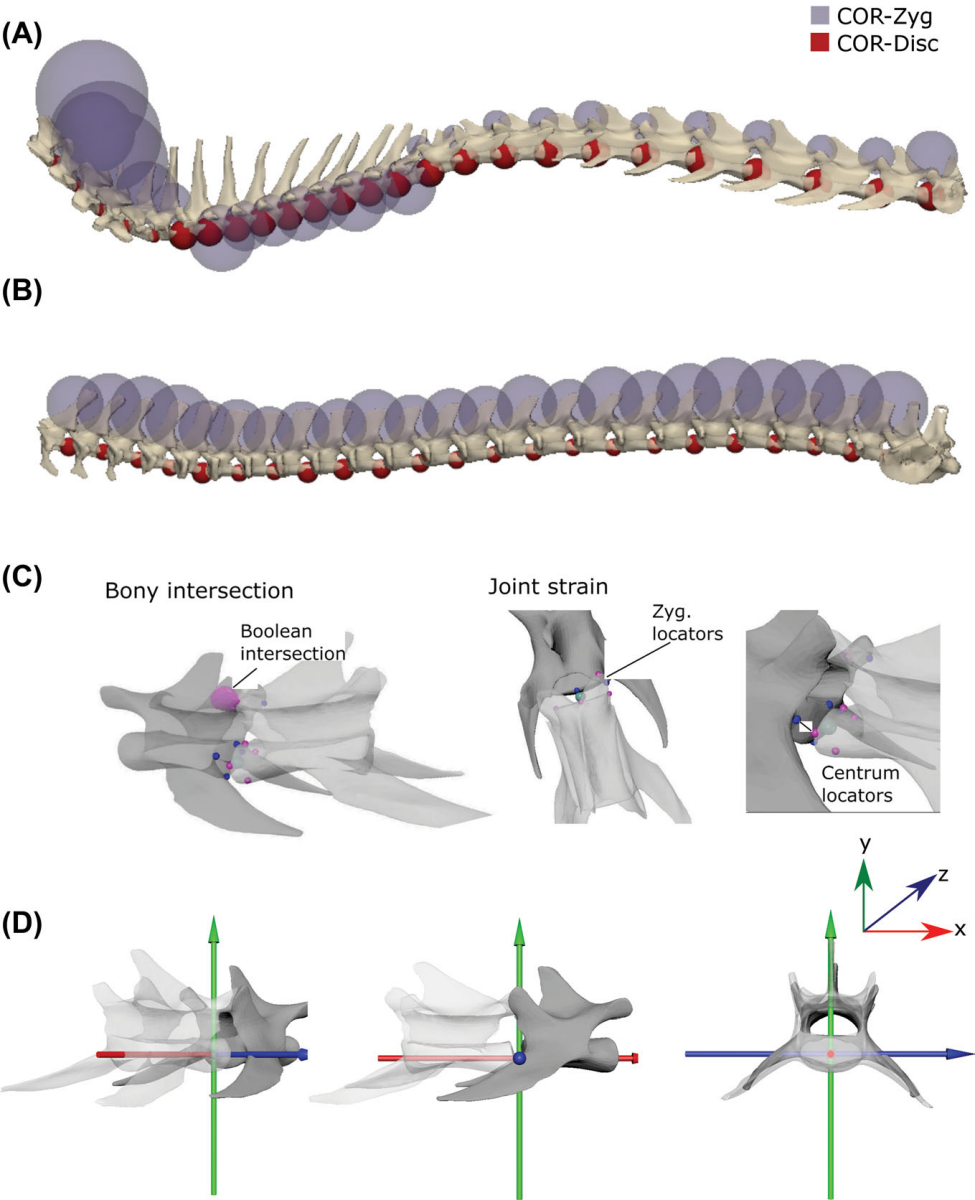
Method for estimating osteological digital range of motion (oROM). Assembled vertebral columns of a cat **(A)** and tegu **(B)**, showing the locations of the center of rotations using the zygapophysis (purple) and disk (red) approach. **(C)** Each motion segment consists of an anterior and posterior vertebra (cat, L5–L6). Landmarks were placed at the centrum extremes (dorsal, ventral, left, right; blue: anterior landmarks; pink: posterior landmarks), as well as the anterior and posterior extremes of the left zygapophysis. Limits on motion are determined by bony intersection between vertebrae, disarticulation of the zygapophyses, and strain at the centra. **(D)** Coordinate system for vertebral bending, where the *x*-axis is axial rotation, *y*-axis lateral bending, and *z*-axis sagittal bending.

Once aligned, vertebral meshes and COR spheres were imported into Autodesk Maya 2019 to create the digital bending model. The coordinate system for each vertebral motion segment (two vertebrae and one intervertebral joint) was established using an axis object from the XROMM Maya Tools (https://bitbucket.org/xromm/xromm_mayatools) that was translated to a locator at the center of the COR sphere for each joint. The axes were rotated manually to their coordinate system position using the interactive rotate tool. The *x*-axis was aligned with the long axis of the vertebral centrum, while the *y*-axis and *z*-axis were aligned with the dorsoventral and mediolateral planes, respectively (Fig. 2D). This coordinate system translates into the following motions: *x*-axis is axial rotation, *y*-axis lateral bending, and *z*-axis sagittal bending.

Anatomical landmarks were manually placed on each joint using the locator tool in Maya to provide reference points for zygapophyseal and centrum strain, as detailed later. Two landmarks were placed on the left zygapophyseal joint at the cranial extreme of the pre-zygapophysis and the caudal extreme of the post-zygapophysis (Fig. 2C). A further eight landmarks were placed on the anterior and posterior centra at the dorsal, ventral, left, and right extremes of the endplates (Fig. 2C).

### AutoBend

Prior to running the digital bending experiment, the posterior vertebra of each motion segment, along with its Boolean object copy and posterior landmark locators, was parented to the COR locator, which was in turn parented to the anterior vertebra, its Boolean copy, and the anterior landmark locators. This was applied using the *set_up_sub_joint* function in AutoBend, in a craniocaudal fashion along the column so that motion at the vertebral joints was hierarchically linked. The experiments were then implemented using the *bending_analysis* function in AutoBend from cranial to caudal. For each motion segment, the posterior vertebra was rotated about the COR locator in half degree increments until a constraint on motion was reached (see later). Vertebrae were rotated in dorsiflexion, ventroflexion, left and right lateroflexion, and left and right axial rotation. When bending stopped, the angular displacement and type of constraint reached was reported. If no constraint was reached, bending was stopped at 45 degrees. The python code for AutoBend is freely available through github (https://github.com/katrinajones/AutoBend).

### Constraints on motion

To estimate intervertebral joint range of motion for each intervertebral joint, three types of motion constraints were considered: bony intersection, zygapophyseal strain, and centrum strain.

Bony intersection was measured by overlap of the anterior and posterior meshes of the vertebral motion segment using a Boolean object. The Boolean object was created in Maya using the Boolean intersection tool, which transforms duplicate meshes into a new object that captures only their overlap. The area of this Boolean object reflects the degree of overlap between the vertebra at a given rotation. For most oROM studies, any overlap area between the vertebrae is a criterion for stopping motion (i.e., herein referred to as an intersection threshold of zero). However, intervertebral joints often fit together very tightly, and motion occurs as joints slide across one another, so even slight unevenness in the surface of the meshes could stop motion prematurely. Therefore, in our model we allow a small intersection threshold before stopping motion to provide additional leeway (see validation and sensitivity analyses later).

Zygapophyseal strain accounts for soft tissue limitations at the synovial joint and was measured using the relative positions of the two zygapophyseal landmarks. In life, the zygapophyseal facet joints have capsular ligaments that control the extent of motion at the joint. These ligaments will prevent overextension of joints during ventroflexion (disarticulation) and dorsiflexion (overlap) (Gál 1993). Here, we model this using a zygapophyseal strain factor—the degree of overlap change permitted between the zygapophyseal facets. Prior to the digital bending experiment, the distance between markers on the caudal tip of the cranial facet and the cranial tip of the caudal facet, along the *x*-axis (craniocaudal component only) was measured at the neutral pose. This served as the reference length for motion during bending. Motion ceased when this distance fell below the lower zygapophyseal strain threshold in ventroflexion (disarticulation) or above the upper threshold in dorsiflexion (overlap, see validation and sensitivity analyses later).

Centrum strain accounts for soft tissue limitations of the intervertebral disk and was measured using the relative positions of the eight centrum landmarks. Intervertebral joints vary in their structure across amniotes, forming an intervertebral disk in mammals and a synovial ball-and-socket joint in most reptiles (Wintrich et al. 2020). In all cases, the outer perimeter of the centrum joint consists of annular ligaments that secure the two vertebrae together. We model the impact of these ligaments and other intervertebral tissues using the centrum strain factor—the degree of distance change permitted between the vertebral centra. Prior to the digital bending experiment, the distance between each pair of landmarks (dorsal, ventral, left, and right) was calculated. Motion ceased during the experiment when this distance exceeded the upper or lower thresholds for centrum strain, depending on the bending direction (see validation and sensitivity analyses later).

### Validation of model assumptions

The AutoBend model relies upon numerous underlying assumptions. To assist with setting up our models, we used the *ex vivo* experimental data to examine several vertebral modeling assumptions that have been applied in the literature and to establish the best modeling conditions for the taxa studied here.

First, we test the impact of the placement of the COR. Estimating the COR is challenging because very little data exists on its location in quadrupedal animals. While the COR may be determined experimentally, for digital oROM approaches to be successfully applied to bone-only specimens it is necessary to determine the approximate location of the COR based on bony anatomy alone. Previous oROM studies have placed the COR in the center of the intervertebral disk or middle of the procoelous ball and socket joint (Molnar et al. 2015; Jurestovsky, Jayne, and Astley 2020), following experimental evidence that suggests it is usually located within the disk (Haher et al. 1991; White III and Panjabi 1990; Selbie, Thomson, and Richmond 1993) and the assumption that a centrally placed COR will minimize deformation of disk soft tissues during bending (COR-disk). COR-disk minimizes shear in the intervertebral disk for the cat, and minimizes interactions between the procoelous centrum ball-and-socket joint for the tegu, by positioning the COR in the center of the intervertebral disk and the center of the procoelous ball joint (caudal aspect), respectively (Fig. 2A and B) (Molnar et al. 2015). However, the zygapophyses have also been implicated as important in determining COR (Schmidt et al. 2008; Penning and Badoux 1987; Kuznetsov and Tereschenko 2010; Belyaev, Kuznetsov, and Prilepskaya 2021). Therefore, we also test an alternative hypothesis that places the COR based on the anatomy of the zygapophyses (COR-zyg). COR-zyg minimizes interactions at the zygapophyseal joints in both taxa by positioning the COR at the center of a sphere fit to the zygapophyseal facets (Fig. 2A and B).

Next, we test the impact of the addition of soft tissue constraints on the estimation of oROM by allowing varying amounts of translation and displacement at the zygapophyses and intervertebral disks. Joint tissues permit small translations during *in vivo* vertebral bending (Haussler et al. 2001) and they have been incorporated into some previous oROM analyses (Molnar et al. 2015; Pierce, Clack, and Hutchinson 2012; Lai, Biewener, and Pierce 2018). Here, we explore the impact of translation on oROM by running analyses with and without translations. For iterations with translation, the posterior vertebra was translated in the direction of bending (e.g., to the left for left lateral bending) if a bony intersection constraint was reached during bending. Joints were translated by 0.5%, 1% and 5% of the square-root vertebral area relative to the neutral position. 0.5% and 1% translation equate to similar magnitudes of translation to those applied in previous studies (i.e., 1.5%/3% of centrum length applied in Molnar et al. 2015) based upon bending in human lumbar joints (Xia et al. 2010). Vertebral area was measured as the average surface area of the meshes of the anterior and posterior vertebra of the joint. Five percent was included to observe the impact of larger degrees of translation on vertebral bending. We calculated the degree of translation based on area not vertebral length because vertebral length is highly variable and therefore area provides a more holistic measure of vertebral size.

Disarticulation of the zygapophyseal joint has been used as a constraint on motion in some oROM studies but the degree of motion permitted is debated. Most commonly, 50% disarticulation is permitted during bending (Molnar et al. 2015; Stevens and Parrish 1999), but experimental data indicate that greater mobility may be possible (Cobley, Rayfield, and Barrett 2013; Kambic, Biewener, and Pierce 2017). To explore this issue further, we ran the AutoBend analysis with and without zygapophyseal constraints, and varied the degree of displacement permitted by +/−50%, +/−75%, and +/−100%. Total disarticulation is considered to be the outer limit of motion because further bending would invoke extreme displacement of the capsular tissues surrounding the joint, while 75% disarticulation most closely matches findings from manipulation of highly mobile turkey necks (Kambic, Biewener, and Pierce 2017). Degree of disarticulation in ventroflexion and degree of overlap in dorsiflexion were measured relative to the neutral starting position of the joint by calculating the relative change in distance between the cranial tip of the caudal zygapophysis and the caudal tip of the cranial zygapophysis (see earlier).

While previous studies have included bony stops and zygapophyseal disarticulation (e.g., Stevens and Parrish 1999), the impact of central tissue in particular has generally been disregarded. Wintrich and colleagues used finite element modeling of individual vertebral joints to assess the degree of bending permitted by the intervertebral disk in plesiosaurs (Wintrich et al. 2019). However, this approach is time-consuming for multiple joints. Therefore, we test a strain-based approach for estimating the effects of central tissue by constraining motion based on the distance between centrum landmarks. We explore this approach by running AutoBend with and without centrum constraints and varying the degree of centrum strain permitted. Experimental data suggests that physiological strain values for human intervertebral disks can range between 13.5% and 57.8% (Costi et al. 2007). Therefore, we test +/−25%, and +/−50% centrum strain, as well as without any centrum constraints.

Finally, we explore the impact of constraints on bony intersection. Usually, no intersection between vertebral meshes is permitted in an oROM analysis. However, some vertebral joints fit together very tightly, which means that small errors in mesh shape or joint articulation can result in small intersections that would stop motion. Therefore, we examined the effect of allowing small amounts of bony intersection versus a strict no-intersection model by varying the intersection threshold. Given that model overlap has not been considered in previous oROM studies, we applied thresholds of 0% (no intersection), 0.25%, 0.5%, and 1% of vertebral area and compared them with the experimental data to ascertain a reasonable starting value for this parameter.

### Sensitivity analyses

To understand the sensitivity of our oROM models to noise associated with model construction and to generate a confidence interval on our estimates of mobility, the automated digital bending experiment was run eight times while three parameters were varied: joint spacing, intersection threshold, and strain. A low and a high value were specified for each to measure the impact of these parameters on the model results.

Joint spacing reflects the relative separation of the two vertebrae by soft tissues and can be difficult to ascertain in bone-only specimens and is thus prone to error in model reconstruction. It was varied by translating the posterior vertebra along the *x*-axis using the *adjust_spacing* function in AutoBend. Spacing was adjusted by 10% relative to the original spacing in the neutral pose, such that the low value was 10% narrower and the high value was 10% wider, reflecting a reasonable degree of error associated with the articulation of the joint based on visual observation.

The intersection threshold reflects the degree of overlap between vertebrae permitted during bending and is included to account for small errors in the reconstruction of the vertebral 3D mesh, such as mesh inflation during wrapping or uneven surfaces. It was calculated as a percentage of the average surface area of the anterior and posterior vertebrae, with a low value of 0.25% and a high value of 0.5% total area, allowing a small degree of intersection during bending.

Finally, placement of the landmarks used to estimate the soft tissue displacements in the model is another potential source of error. Landmarking error will result in inaccuracies in the estimation of zygapophyseal and centrum strain, so we artificially vary the strain values by 10% to allow for slightly increased or decreased displacement. We apply strain of −45/−55% (lower) and +45/+55% (upper), relative to the neutral position, to both centrum and zygapophyses in the sensitivity analysis.

### oROM data analysis

The bending data for all six bending directions (degrees of motion and constraint type) were imported into R (R Development Core Team 2009) for analysis. Left- and right-side measures were averaged to account for any asymmetry then doubled to estimate total lateral and axial mobility. Ventroflexion and dorsiflexion were summed to provide a measure of overall sagittal bending. This resulted in three directions of motion for most analyses: lateral, sagittal, and axial. Dorsiflexion and ventroflexion were analyzed separately when examining which constraints stopped motion because they tended to differ. Patterns of oROM in different bending directions were visually compared with the experimental ROM obtained from the original biomechanical study (Jones et al. 2020). The impact of biological and sensitivity parameters on oROM for each species was measured using an analysis of variance (ANOVA), with degrees of motion as the response variable, and the main effects joint, bending direction, their interaction, and the sensitivity parameters coded as high or low.

### Estimating intervertebral joint stiffness

Vertebral joint function is determined not only by total mobility but also by joint stiffness—the moment required to induce bending (Koob and Long 2000; Long et al. 1997; Molnar, Pierce, and Hutchinson 2014; Oliver et al. 2016). Previous studies estimating vertebral function have either modeled the vertebral column as a beam resisting bending (Slijper 1946; Jones et al. 2021), or by estimating the lever arms and/or forces that are generating the bending moment *in vivo* (Christian and Preuschoft 1996; Pierce, Clack, and Hutchinson 2011). However, joint stiffness is the angular displacement achieved for a given moment applied. Therefore, we propose a new metric based on the estimated bending moment and oROM, a proxy for maximum angular displacement (Fig. 2B), using the following equation.


$$\begin{equation*}{\rm{Stiffness}} = \frac{{\left( {{\rm{Force\ }} \times {\rm{Moment\ arm}}} \right)}}{{{\rm{oROM}}}}\end{equation*}$$

Estimating the moment arm for bending from muscular forces applied *in vivo* is very difficult. Further, during the passive validation experiments used here the joints reached their maximum angular displacement (equivalent to oROM) when soft tissues were able to fully resist the load applied, not due to *in vivo* muscle loadings. Therefore, to model stiffness in this context, we estimate the moments for resisting bending during passive loading, rather than the bending moments applied during life. Lesion experiments in mammals have shown that the intervertebral disks, ligamenta flava, and capsular ligaments are most important for passively resisting sagittal bending in the vertebral column (Gál 1993). Furthermore, in the digital bending experiments, sagittal motion was most frequently halted by tension or compression in the zygapophyseal joint (see results). Therefore, we use the distance from the COR-disk to the top of the vertebral arch (approximate location of ligamenta flava and joint capsule) to estimate lever arms in resisting sagittal bending, which was estimated from published linear measures as half centrum height plus vertebral arch height (Jones et al. 2020). Lateral bending was most frequently resisted by tension or compression in the centrum joint (see results), therefore the lever arm was taken as half the width of the centrum. Following previous studies, force was approximated using the cross-sectional area of the centrum (Christian and Preuschoft 1996), scaled to the power 3/2.

For example, for a joint with averaged posterior and anterior centrum dimensions of 8 mm height and 10 mm width, vertebral arch height of 3 mm, and estimated oROM of 15 degrees in sagittal bending and 12 degrees in lateral bending, joint stiffness would be estimated as follows.


$$\begin{eqnarray*}{\textit {Stiffness}}\ \left( {Sagittal} \right) &=& \ \frac{{{{\left( {8 \times 10} \right)}^{\frac{3}{2}}}\ \times \left( {\left( {\frac{8}{2}} \right) + 3} \right)}}{{15}}\nonumber\\ &=& \frac{{716\ \times 7}}{{15}} = 334\end{eqnarray*}$$

$$\begin{eqnarray*}{\textit {Stiffness}}\ \left( {Lateral} \right)\ &=& \ \ \frac{{{{\left( {8 \times 10} \right)}^{\frac{3}{2}}}\ \times \left( {\frac{{10}}{2}} \right)}}{{12}}\nonumber\\ &=& \frac{{716\ \times 5}}{{12}} = \ 298\end{eqnarray*}$$

Units are mm^4^ per degree but the values are not meaningful because the metric is intended to be used in a purely comparative context. Estimated stiffness was then logged and scaled by mean centrum length for each species to account for their size difference.

## Results and discussion

### Validation of model assumptions

**Fig. 3:**
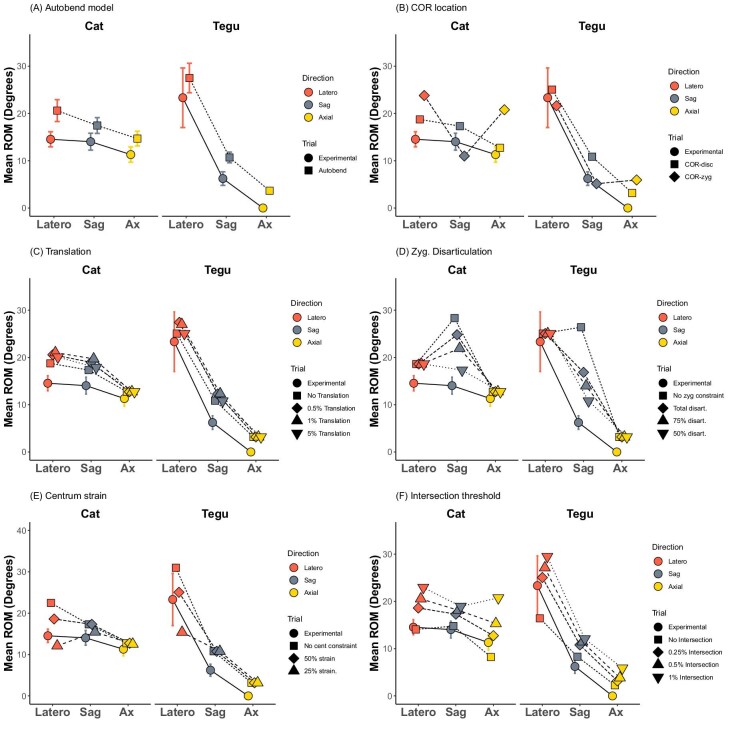
Summary of validation results showing relationships between directions of mobility in mean ROM. Comparison of experimental data with AutoBend model under various modeling parameters. **(A)** Final model with optimal parameters, **(B)** COR location, **(C)** degree of translation, **(D)** amount of zygapophyseal disarticulation, **(E)** amount of centrum strain, and **(F)** degree of intersection permitted. Range of motion is averaged along the column in each bending direction. Lat: lateral bending, Sag: sagittal bending, Ax: axial rotation.

**Fig. 4:**
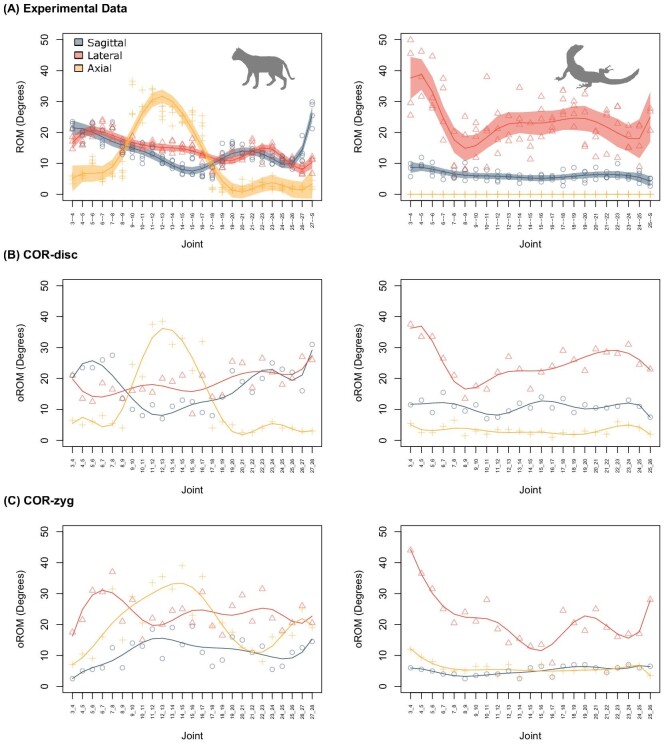
Impact of center of rotation (COR) location on along-column patterns of mobility. **(A)** Experimental data. Shaded region: 95% confidence interval. **(B)** AutoBend run with the COR located within the disk or procoelous ball and socket joint. **(C)** AutoBend run with COR located between the zygapophyseal joints. Results for translation, centrum strain, and intersection threshold are provided in the Supplementary Material.

**Fig. 5:**
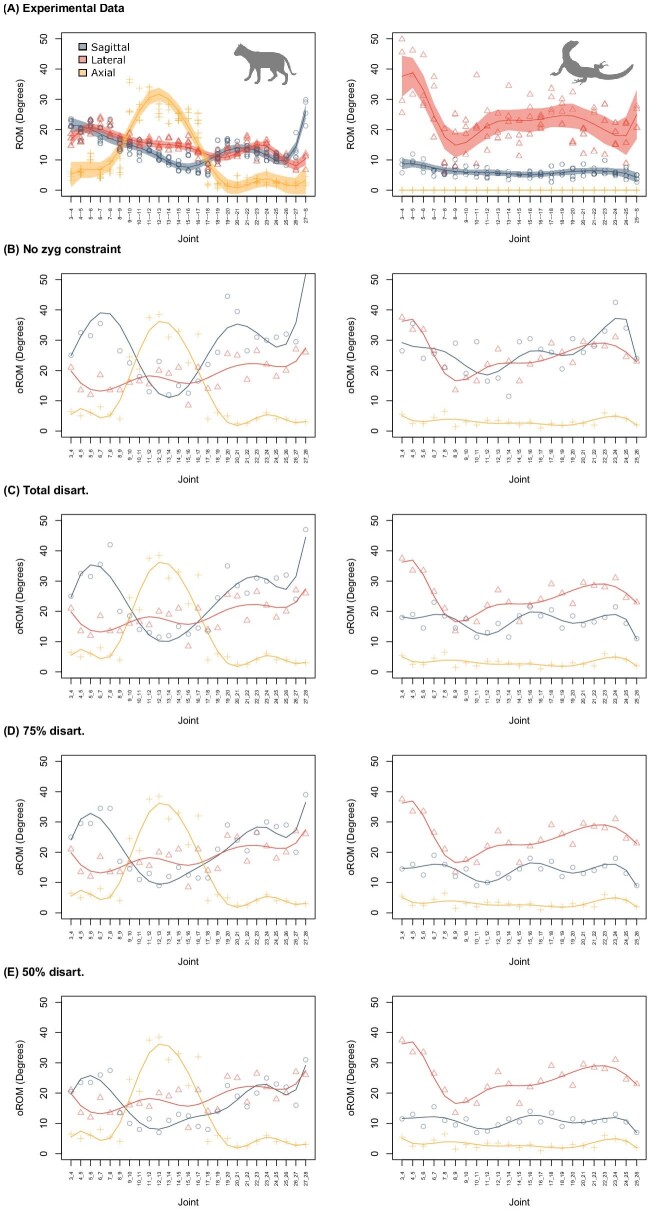
Impact of zygapophyseal disarticulation on along-column patterns of mobility. **(A)** Experimental data. Shaded region: 95% confidence interval. **(B)** AutoBend with no zygapophyseal constraints. AutoBend with **(C)** 100%, **(D)** 50%, and **(E)** 25% strain permitted at the zygapophyseal joint. Results for translation, centrum strain, and intersection threshold are provided in the Supplementary Material.

Constructing a digital oROM model requires numerous assumptions, but the paucity of biomechanical data from the vertebral column has presented a barrier to testing their validity. Here, we examined five of the fundamental underlying assumptions of oROM modeling to examine their impact on model outcomes and refine best practices (Jones et al. 2020) (Figs. 3–5; Figs. S1–S3). Specifically, we examined location of the COR, the degree of joint translation allowed, the impacts of zygapophyseal and central soft tissues, and the degree of mesh intersection.

When digitally articulating a vertebral motion segment for oROM analysis, the position of the COR about which bending occurs must be determined. Data on the location of the vertebral COR in quadrupedal animals are rare (but see Inoue et al. 2017; Selbie, Thomson, and Richmond 1993; Penning and Badoux 1987), and estimates in humans vary depending on measurement technique (Samagh et al. 2011). Experimental work most commonly finds the COR to be somewhere within the intervertebral disk (Haher et al. 1991; Selbie, Thomson, and Richmond 1993), vertebral arch (Samagh et al. 2011), or at the edge of the posterior vertebral body when the centrum endplates are curved (Penning and Badoux 1987). Based on this, and the assumption that a disk-located COR will minimize strain in disk tissues, some previous oROM studies have placed the COR at the inferred center of the disk or procoelous ball and socket joint (Molnar et al. 2015; Jurestovsky, Jayne, and Astley 2020; Stevens and Parrish 2005). Although the location of the COR is not explicitly defined in many studies (e.g., Vidal et al. 2020; Taylor and Wedel 2013), it is assumed they have also followed this convention. However, the morphology of the zygapophyses has also been hypothesized to guide intervertebral movements (Schmidt et al. 2008; Penning and Badoux 1987; Kuznetsov and Tereschenko 2010; Belyaev, Kuznetsov, and Prilepskaya 2021), so we explore the impact of allowing their morphology to determine the location of the COR.

We test the standard COR-disk location against COR-zyg, which is located to minimize the interaction between the zygapophyseal facets. We determined the optimal position for COR-zyg by placing it at the center of a sphere determined by the arc of the zygapophyseal facets (Fig. 2), using a similar approach to that applied by Belyaev et al. (2021) in two dimensions for determining vertebral mobility from zygapophyseal anatomy in mammals. Comparing oROM patterns from both models to experimentally derived ROM (Jones et al. 2020) (Figs. 3B and 4) reveals that the COR-disk produced much more realistic oROM patterns than COR-zyg. The COR-disk model successfully recreated the relative patterns (Fig. 4B) and magnitudes (Fig. 3B) of intervertebral joint bending in both the cat and the tegu. By contrast, the COR-zyg model overestimates lateroflexion in both species and does not recover overall craniocaudal trends in intervertebral joint mobility (Fig. 4C). This suggests the true COR is likely located within the disk, and supports previous studies that have used a centrum located COR (Molnar et al. 2015; Jurestovsky, Jayne, and Astley 2020).

Soft tissues surrounding intervertebral joints may allow small amounts of translation to occur during bending without destabilizing the joint (Haussler et al. 2001; Xia et al. 2010). While most vertebral oROM models have not included translations (e.g., Taylor and Wedel 2013; Vidal et al. 2020; Stevens and Parrish 2005), Molnar et al. (2015) included translations of 1.5% and 3% centrum length based upon magnitudes of translation occurring in human lumbar joints (Xia et al. 2010). To examine the impact of translations on vertebral bending, we compared models with no translations to those that permitted 0.5%, 1%, and 5% translation. This was measured as a proportion of square-root vertebral area, rather than against vertebral length, to provide a fairer comparison across taxa with variable vertebral elongation. Half a % translation measured in this way produces similar magnitudes of translation to the 1.5% centrum length used in the Molnar et al. (2015) study. However, we found that including translations had very little effect on the AutoBend model (Fig. 3C; Fig. S1). As degree of translation was increased there was a slight increase in mobility in lateroflexion in the posterior dorsal joints of the tegu (Fig. 3C; Fig. S1). However, once translations became very high (5%), mobility decreased again (Fig. 3G; Fig. S1D). This is likely due to joint disarticulation or misalignment caused by the large translations and suggests this value is too high. The effect of translation is likely minimized in AutoBend because soft tissue constraints were applied in addition to bony intersection, thereby preventing excessive rotations. Given these minimal effects, we exclude translations from our model going forward.

The role of zygapophyseal soft tissues in constraining vertebral motion in digital models has also been hotly debated. Early work by Stevens and Parrish (1999) permitted sagittal bending at the intervertebral joint only to the point at which 50% disarticulation/overlap of the zygapophyseal facets was achieved, but greater amounts of mobility have been observed in cadaveric studies (Kambic, Biewener, and Pierce 2017; Cobley, Rayfield, and Barrett 2013; Stevens and Parrish 2005; Taylor, Wedel, and Naish 2009). We explicitly test the impact of zygapophyseal soft tissues by comparing a model without any zygapophyseal constraints to those that permit varying degrees of displacement. The more broadly applied 50% disarticulation assumption was compared with two less conservative models that allow 75% and 100% disarticulation, respectively. In the model that excluded zygapophyseal constraints, sagittal bending was strongly overestimated and the craniocaudal patterns from the experimental data were not recovered (Jones et al. 2020) (Figs. 3D and 5B). This suggests that including the impact of zygapophyseal soft tissues is very important for estimating oROM. Of the models that included a zygapophyseal constraint, 50% disarticulation most closely replicates the magnitudes of sagittal bending obtained in the cadaveric experiments (Fig. 3D), with 75% and 100% disarticulation overestimating bending in the sagittal direction compared with lateroflexion and axial twisting (Fig. 5). In cadaveric experiments on bird necks, values of disarticulation closer to 75% were obtained (Kambic, Biewener, and Pierce 2017; Cobley, Rayfield, and Barrett 2013). This discrepancy may relate either to the extreme mobility of bird necks, or to differences in the way that overlap was measured. For example, Kambic and colleagues measure overlap relative to total facet length (Kambic, Biewener, and Pierce 2017). However, zygapophyseal facets often do not totally overlap and may vary in shape, size and orientation, so we measure overlap changes relative to the overlap at the neutral starting position, which reflects the maximum achieved while the joint is in good articulation.

While the impact of zygapophyseal tissues has been explored in previous work (see earlier), only one previous study has attempted to incorporate intercentrum soft tissues into vertebral oROM (Wintrich et al. 2019). They modeled the intervertebral disk using finite element analysis (FEA) that limited motion based on the stress produced in the disk. However, there are several limitations to this approach. FEA models are time consuming to construct and run when examining many sets of vertebral joints, and given the uniform distribution of material properties applied, the results are likely to be similar to those obtained from examining pure displacement. By contrast, the strain-based approach here is rapid to implement as it relies on only a few landmarks, and can be conducted within Maya in concert with other important constraints such as bony intersection and zygapophyseal overlap. We tested the impact of centrum displacement on oROM estimation by comparing models without centrum constraints to those that permitted varying degrees of strain in the disk, estimated based on the displacement of centrum landmarks (Fig. 3E; Fig. S2). Results reveal that excluding centrum constraints leads to considerable overestimation of lateroflexion relative to the experimental data (Fig. 3E), which suggests that including the intercentral soft tissues has the potential to improve oROM models. Restricting movement at 50% strain most closely replicated the experimental data in terms of the relative mobility in each bending direction (Fig. 3E; Fig. S2C), while 25% strain was overly restrictive, and underestimated lateroflexion in the tegu (Fig. 3E; Fig. S2D).

Finally, we explored the impact of varying the amount of mesh overlap permitted during bony intersection. Traditionally, bending ceases as soon as the meshes touch, but permitting a small amount of intersection allows leeway in the model when estimating bony stops. This is particularly important in vertebrae because they are complex and tightly interlocking 3D joints that are susceptible to overlap due to minor mesh or alignment errors. Results demonstrate that when intersection threshold is set to 0%, under a strict no-intersection model, mobility is underestimated in lateroflexion and axial rotation (Fig. 3F; Fig. S3B). This may relate to unevenness in mesh surfaces or to slight misalignment, and suggests that incorporating a small amount of intersection into oROM models can be helpful for reconstructing motion. Conversely, allowing 1% intersection strongly overestimated mobility, suggesting that this value is excessive (Fig. 3F; Fig. S3E). Instead, both 0.25% and 0.5% intersection produced realistic values of vertebral bending (Fig. 3F; Fig. S3C and D), and so both are explored in the sensitivity analyses later.

### oROM estimation by AutoBend

**Fig. 6:**
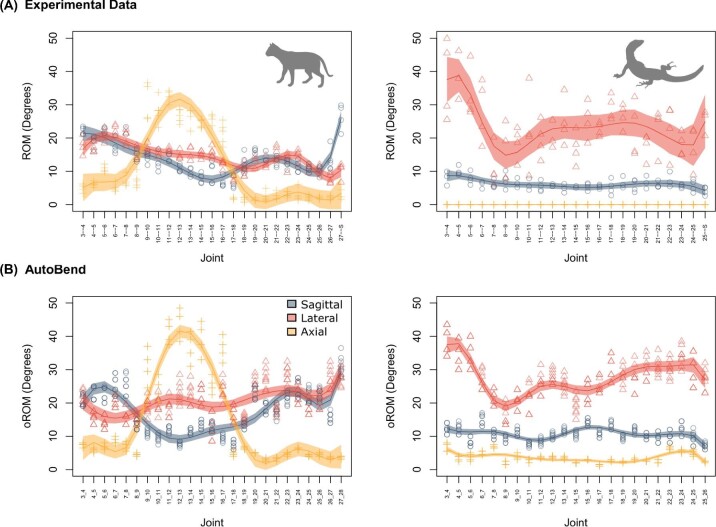
Comparison of experimental ROM and automated digital bending in AutoBend using optimal parameters. **(A)** Experimental ROM data derived from cadaveric bending experiments. **(B)** oROM from AutoBend estimated with COR-disk and no translation permitted. The model was run eight times while varying joint spacing (+−10%), intersection threshold (0.25%/0.5%), and zygapophysis/centrum strain (+−45%/55%) to consider sensitivity to model construction (see Sensitivity Analysis). Shaded regions indicate 95% confidence interval.

Based on the parameter validation earlier, we ran the AutoBend model with the COR location within the intervertebral disk and no translation permitted. Further, we varied the joint spacing by 10%, the intersection threshold (0.25/0.5%), and the degree of centrum and zygapophysis strain (+−45%/55%) to estimate errors associated with model construction (see Sensitivity Analysis). Comparing the AutoBend model outputs with experimental data for the cat and tegu revealed a strong correspondence in both craniocaudal patterns and overall magnitudes of motion for both taxa (Jones et al. 2020) (Fig. 6). Key components of the biological variation were recovered, including the dramatic peak in axial rotation in the thoracic region of the cat, and the high mobility in lateroflexion in the neck of the tegu (Fig. 6B). Further, the relative proportions of bending in different directions were recovered, though absolute values of average motion were overestimated by around 5 degrees in the cat (Fig. 6B). These data suggest that AutoBend represents an improvement in axial oROM estimation because previous validations have recovered only a loose correspondence between experimental and digital data (Molnar et al. 2015) or no correlation at all (Cobley, Rayfield, and Barrett 2013). In particular, the addition of new model parameters to account for the impact of soft tissues and mesh overlap significantly improved the model. Further, by automating the data-collection step, this approach offers greater repeatability, time efficiency, and enhanced potential for the exploration of parameter spaces associated with oROM modeling in the future.

### Limits on intervertebral joint mobility

**Fig. 7:**
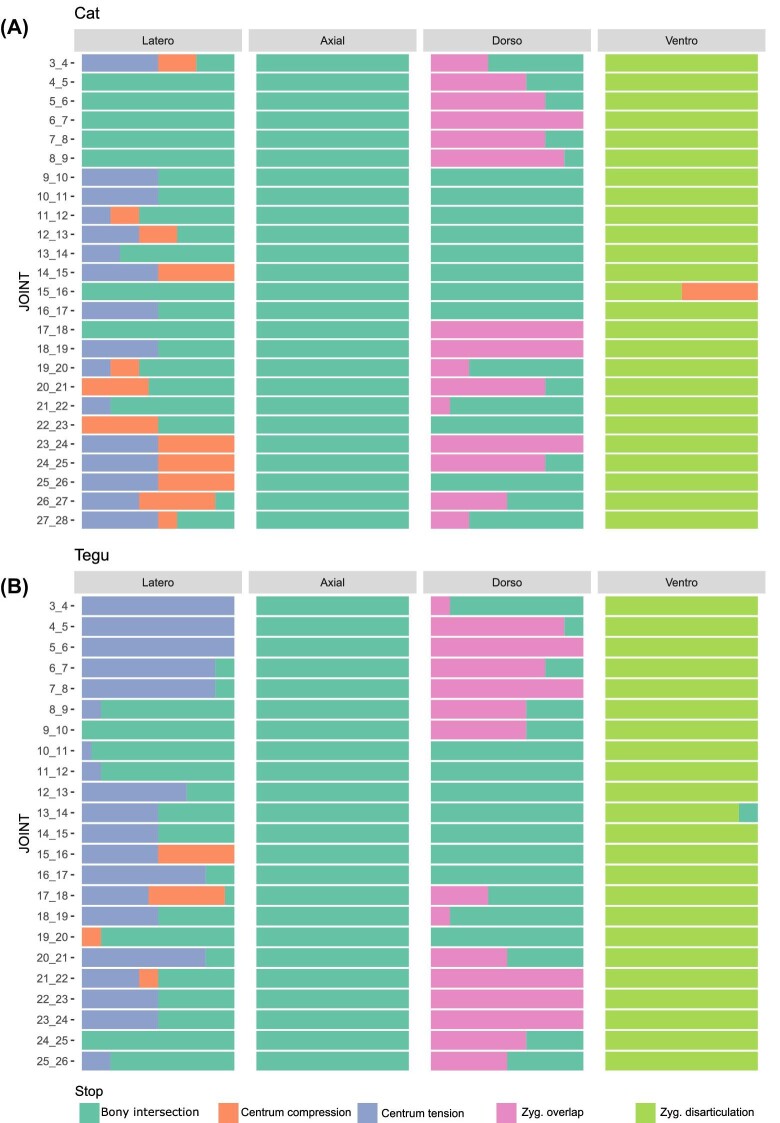
Constraints on oROM. Prevalence of different constraints in stopping motion for each joint and bending direction in **(A)** cat and **(B)** tegu, calculated across the sensitivity repeats. Latero: lateroflexion; Axial: axial rotation; Dorso: dorsiflexion; Ventro: ventroflexion. Bony intersection is most important in restricting axial rotation, while zygapophyseal disarticulation restricts ventroflexion. Dorsiflexion is resisted by both zygapophyseal overlap and bony intersection, while lateroflexion is resisted by bony intersection and centrum constraints.

To further investigate the role that different constraints played in estimating oROM, we determined the type of constraint that stopped motion for each joint for axial rotation and lateroflexion, as well as dorsiflexion and ventroflexion separately (Fig. 7). These data were gathered for each of the vertebral joints and eight repeats of the AutoBend analysis and are represented as stacked bar charts (Fig. 7). Bony intersection is the only constraint limiting axial rotation in both species because the zygapophyseal joints sit in close articulation with one another, rapidly intersecting during twisting for most joints. This supports lesion experiments in which removal of the vertebral arches results in the greatest increase in axial rotation in human lumbar joints (Heuer et al. 2007). An exception is the anterior dorsal joints in the cat (T2–10), where horizontally oriented pre-diaphragmatic joints allow vertebrae to slide pass one another during axial rotation, only intersecting when reaching the lateral arch (Jones et al. 2020). Lateroflexion was limited either by bony intersection or centrum strain (Fig. 7). Centrum compression results from the centrum landmarks on the inside of the lateral bend approaching each other, while centrum tension results from the landmarks on the outside of the lateral bend moving too far apart. Similarly, in human lumbar joints, lesion of the annulus portion of the intervertebral disk has the most significant impact on lateroflexion (Heuer et al. 2007). In contrast, zygapophyseal strain was the most important determinant of sagittal bending. In ventroflexion, zygapophyseal disarticulation primarily constrains motion, while constraints were mixed between zygapophyseal overlap and bony intersection between the zygapophyses in dorsiflexion. Thus, morphology of the zygapophyseal joint appears to be a key determinant of sagittal bending in the cat and tegu. This supports previous experimental work on lumbar joints in a range of mammals, in which sequential lesion of tissues revealed that zygapophyseal overlap primarily resists dorsiflexion, while the ligamenta flava that run between the vertebral arches are a key component of ventroflexion resistance (Gál 1993).

### Sensitivity analysis

**Fig. 8:**
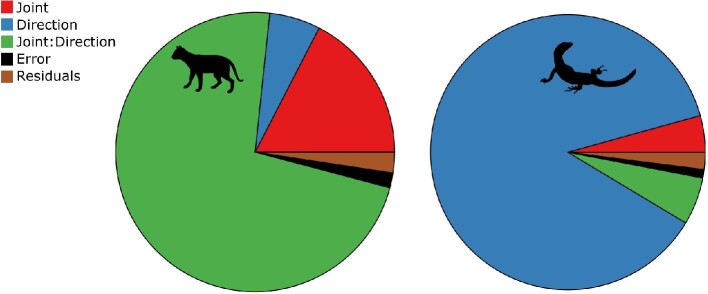
Sensitivity of approach to model parameters. Effect sizes (sums of squares) of model parameters from an ANOVA to determine the sensitivity of oROM estimates to errors associated with: joint spacing, intersection threshold, and joint strain (centrum and zygapophyses). Effect sizes of these parameters (black) are very small compared with main effects of direction of motion and joint, suggesting that the analysis is robust to these assumptions. See Table S1 in the Supplementary Material for detailed ANOVA results.

To account for errors that might arise during model construction and provide confidence intervals for our estimates of mobility, we conducted a sensitivity analysis focused on three potential sources of error: variations in intersection threshold associated with mesh generation and alignment errors, joint spacing errors, and variations in zygapophyseal and centrum strain associated with errors in landmark placement. An ANOVA comparing the impact of these parameters with biological variation in both species revealed that although all effects are highly significant (Table S1, *P* < 0.001), the sensitivity parameters explain only a minor component of the total variation in oROM in both species (Table S1, effect size: sums of squares; Fig. 8). Instead, oROM variation in the cat is primarily explained by the interaction between joint and bending direction (Fig. 8), reflecting the high degree of along-column variation in bending patterns (Fig. 6). In the tegu, most variation was explained by bending direction only (Fig. 8), reflecting the emphasis on lateroflexion relative to other bending directions in lizards and their relative uniformity along the column (Fig. 6). Our data show that intervertebral joint range of motion can be reasonably estimated from bone-only digital models by applying multiple constraints on motion that are based on the vertebral anatomy and function. Further, the sensitivity analyses presented demonstrate that small errors in model construction are likely to have little impact on mobility estimation, generating variation an order of magnitude lower than the biological variation (Fig. 8).

### Intervertebral joint stiffness

Despite the importance of maximum range of motion to vertebral joint function, joint stiffness also plays a critical role (Koob and Long 2000; Nowroozi et al. 2012). Unfortunately, estimating stiffness from morphology alone requires a bigger theoretical leap than oROM, which can be measured empirically. Joint stiffness is the bending moment required to produce a given amount of angular displacement, with stiffer joints requiring greater input moment to impart motion than compliant ones (Knudson 2007). While the moment applied to the joint during the static bending experiments cannot be estimated outside the context of the experiment (Jones et al. 2020), it is possible to estimate the moment resisting bending at maximum ROM when the joint is in static equilibrium. In particular, the lever arm for resisting bending depends on the location of the soft tissue structures most critical in generating resistance, which can be determined through lesion experiments and by examining the constraints on motion in the digital bending experiments. Based on these data, we determined that the intercentral soft tissues (annular ligaments) are the most important for resisting lateroflexion, while vertebral arch tissues (capsular ligaments, ligamenta flava) played a large role in resisting sagittal bending (Gál 1993; Heuer et al. 2007) (see earlier). Therefore, we estimated the moment resisting bending at the maximum range of motion using the lever arm of these structures, which equates to the distance from the COR-disk (middle of disk) to the lateral extreme of the centrum and top of the vertebral arch, respectively. Centrum area has been used as a proxy for maximum loading in the vertebral column (Christian and Preuschoft 1996; Slijper 1946) and was used a proxy for force when estimating moment.

**Fig. 9:**
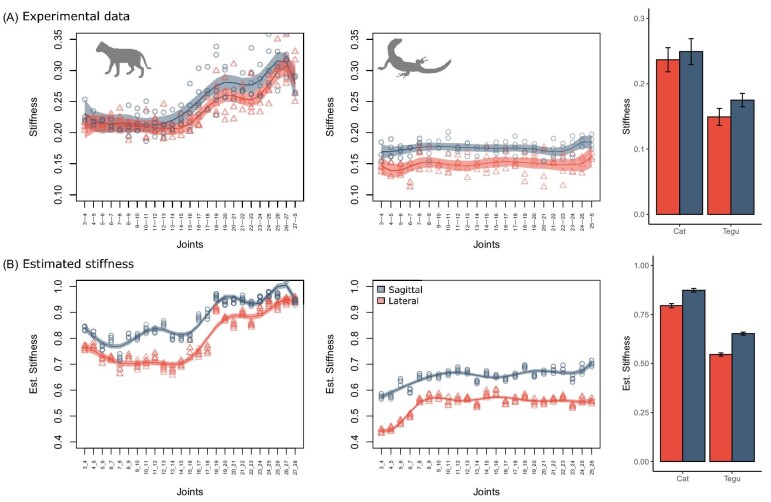
Estimation of along-column stiffness from oROM. **(A)** Stiffness measured from cadaveric bending experiments in cat and tegu**. (B)** Stiffness estimated from oROM and vertebral morphology. Bar charts indicate whole column mean stiffness. Shaded areas indicate 95% confidence intervals.

Our stiffness model can successfully recover craniocaudal and between-species patterns in joint stiffness (but not magnitudes) in both the cat and tegu (Fig. 9). Four major trends are evident. First, the stiffness model accurately recovers the increase in stiffness in the posterior dorsal column in the cat that is not present in the tegu (Fig. 9A). Second, the stiffness model reconstructs successfully a more compliant neck in the tegu (Fig. 9B). Third, the model recovers the observed pattern of greater stiffness in sagittal than lateral bending in both taxa. Finally, it correctly reconstructs higher overall mean stiffness in the cat than in the tegu (Fig. 9, bar charts). Therefore, despite differing magnitudes, the stiffness model proposed here seems to provide a reasonable estimate of stiffness patterns in the vertebral column for both taxa and thus has great potential for inferring axial function from bone-only models, including those from extinct taxa.

### Recommendations and limitations

The combination of starting parameters applied here (centrum-located COR, no translation, ∼0.5 centrum and zygapophysis strain, 0.25–0.5% intersection threshold) seems to be a reasonable estimate for real constraints on the vertebral column in mammals and reptiles because the magnitudes of mobility estimated here are close to those obtained from cadaveric experiments (Fig. 6). Considering the extreme morphological and functional differences, as well as the phylogenetic distance between our study taxa, we suggest that these parameters may be a good starting point for applying the automated bending method in the future. Nonetheless, model parameters in AutoBend are fully controlled by the user, offering the potential to fine-tune the model to other taxonomic groups where validation data are available (Molnar, Pierce, and Hutchinson 2014; Kambic, Biewener, and Pierce 2017). We therefore recommend that the parameters are validated against experimental data, where possible, when AutoBend is applied to a new study system, especially if the vertebral morphology and function are considerably distinct than presented here or to better constrain fossils that fall within a different phylogenetic bracket.

As with all models, AutoBend has its limitations. First, the current implementation examines each bending direction separately and does not account for coupled motions. Coupled motions are thought to occur in the spine (Legaspi and Edmond 2007; Liebsch and Wilke 2018; Kingma et al. 2018), although their prevalence during *in vivo* behavior is poorly understood. AutoBend could be easily modified to incorporate coupled motions in future work, but its application is limited by the large number of axis combinations that would be required to fully explore mobility space. Further, experimental data incorporating coupled motions are currently unavailable, so it would be difficult to validate the results of a coupled AutoBend model. Second, the strain-based soft tissue modeling applied here assumes uniform material properties of tissues across vertebral joints, bending directions, and species. Future studies could vary the strain factors applied to account for known variations in material properties if those data were available. Despite these simplifications of a very complex system, AutoBend reflects a significant step forward in estimating intervertebral joint mobility, and may provide useful insights into broad, cross-taxonomic patterns when applied in a comparative context.

## Conclusion

The axial skeleton is a critical component of the vertebrate locomotor apparatus, but limited data on vertebral form–function relationships have hindered our understanding of axial evolution. *In vivo* vertebral motions are difficult to measure because the vertebral column consists of multiple small elements that must be visualized through the tissues of the trunk, presenting a significant challenge for X-ray imaging during movement. Therefore, data on axial function have been limited to select model taxa that can be easily manipulated in the lab (Schilling and Hackert 2006; Wachs, Fischer, and Schilling 2016; Kambic, Biewener, and Pierce 2017). Here, we have presented an automated method called AutoBend for digitally estimating intervertebral oROM and stiffness in bone-only models, which can be derived from museum specimens or the increasingly widely available CT data in online repositories (e.g., MorphoSource). This automated and parameterized oROM approach offers greater repeatability and is far more time efficient than previous methods that relied upon manual manipulation of digital models (e.g., Molnar et al. 2015; Stevens and Parrish 1999). We have validated this approach using experimental data and demonstrated that it can reasonably reconstruct mechanical function in two extant amniotes, a domestic cat and an Argentine black and white tegu. This validation in a mammal and a lizard offers enormous potential for application of these methods to other amniotes (or vertebrates broadly), including both extant and fossil taxa. Further validation of the approach on other extant taxa in the future has the potential to expand its applicability, and to hone the model parameters to individual groups. Therefore, AutoBend provides a very promising avenue for future research reconstructing vertebral function in difficult-to-study organisms (e.g., non-model extant species or fossils) and can thus contribute to a broader understanding of the evolution of vertebral function.

## Supplementary Material

obab026_Supplemental_Files

## Data Availability

The data underlying this article are available in Harvard Dataverse at https://doi.org/10.7910/DVN/XP3JVZ, and can be accessed with DOI 10.7910/DVN/XP3JVZ. Python and R code can be found on github (https://github.com/katrinajones/AutoBend).

## References

[bib1] Arnold P , Fischer MS, Nyakatura JA. 2014. Soft tissue influence on *ex vivo* mobility in the hip of Iguana: comparison with *in vivo* movement and its bearing on joint motion of fossil sprawling tetrapods. J Anat 225:31–41. 24762236 10.1111/joa.12187 PMC4089344

[bib2] Belyaev RI , Kuznetsov AN, Prilepskaya NE. 2021. A mechanistic approach for the calculation of intervertebral mobility in mammals based on vertebrae osteometry. J Anat 238:113–30. 32951205 10.1111/joa.13300 PMC7754917

[bib3] Boyer DM , Gunnell GF, Kaufman S, McGeary TM. 2016. MorphoSource: archiving and sharing 3-D digital specimen data. Paleontol Soc Pap 22:157–81.

[bib4] Brainerd EL , Owerkowicz T. 2006. Functional morphology and evolution of aspiration breathing in tetrapods. Respir Physiol Neurobiol 154:73–88. 16861059 10.1016/j.resp.2006.06.003

[bib5] Buchholtz EA . 1998. ‘Implications of vertebral morphology for locomotor evolution in early Cetacea.’ In: The emergence of whales. Boston, MA: Springer.

[bib6] Carrier DR . 1987. The evolution of locomotor stamina in tetrapods: circumventing a mechanical constraint. Paleobiology 13:326–41.

[bib7] Christian A , Preuschoft H. 1996. Deducing the body posture of extinct large vertebrates from the shape of the vertebral column. Palaeontology 39:801–12.

[bib8] Cieri RL , Hatch ST, Capano JG, Brainerd EL. 2020. Locomotor rib kinematics in two species of lizards and a new hypothesis for the evolution of aspiration breathing in amniotes. Sci Rep 10:1–10. 32398656 10.1038/s41598-020-64140-y PMC7217971

[bib9] Cobley MJ , Rayfield EJ, Barrett PM. 2013. Inter-vertebral flexibility of the ostrich neck: implications for estimating sauropod neck flexibility. PLoS One 8:e72187. 23967284 10.1371/journal.pone.0072187 PMC3743800

[bib10] Costi JJ , Stokes IA, Gardner-Morse M, Laible JP, Scoffone HM, Iatridis JC. 2007. Direct measurement of intervertebral disk maximum shear strain in six degrees of freedom: motions that place disk tissue at risk of injury. J Biomech 40:2457–66. 17198708 10.1016/j.jbiomech.2006.11.006 PMC1955952

[bib11] Dickson BV , Clack JA, Smithson TR, Pierce SE. 2021. Functional adaptive landscapes predict terrestrial capacity at the origin of limbs. Nature 589:242–5. 33239789 10.1038/s41586-020-2974-5

[bib12] Gál JM . 1993. Mammalian spinal biomechanics 2: intervertebral lesion experiments and mechanisms of bending resistance. J Exp Biol 174:281–97. 8440969 10.1242/jeb.174.1.281

[bib13] Gatesy SM . 1991. Hind limb movements of the American alligator (*Alligator mississippiensis*) and postural grades. J Zool 224:577–88.

[bib14] Haher TR , Bergman M, O'Brien M, Felmly WT, Choueka J, Welin D, Chow G, Vassiliou A. 1991. The effect of the three columns of the spine on the instantaneous axis of rotation in flexion and extension. Spine 16:S312–8. 1785078

[bib15] Haussler KK , Bertram JEA, Gellman K, Hermanson JW. 2001. Segmental *in vivo* vertebral kinematics at the walk, trot and canter: a preliminary study. Equine Vet J 33:160–4. 10.1111/j.2042-3306.2001.tb05381.x 11721560

[bib16] Hedrick BP , Mason Heberling J, Meineke EK, Turner KG, Grassa CJ, Park DS, Kennedy J, Clarke JA, Cook JA, Blackburn DC. 2020. Digitization and the future of natural history collections. Bioscience 70:243–51.

[bib17] Heuer F , Schmidt H, Klezl Z, Claes L, Wilke H-J. 2007. Stepwise reduction of functional spinal structures increase range of motion and change lordosis angle. J Biomech 40:271–80. 16524582 10.1016/j.jbiomech.2006.01.007

[bib18] Hutchinson JR , Garcia M. 2002. Tyrannosaurus was not a fast runner. Nature 415:1018–21. 11875567 10.1038/4151018a

[bib19] Hutson JD , Hutson KN. 2015. Inferring the prevalence and function of finger hyperextension in Archosauria from finger-joint range of motion in the American alligator. J Zool 296:189–99.

[bib20] Hutson JD , Hutson KN. 2012. A test of the validity of range of motion studies of fossil archosaur elbow mobility using repeated-measures analysis and the extant phylogenetic bracket. J Exp Biol 215:2030–8. 22623191 10.1242/jeb.069567

[bib21] Hutson JD , Hutson KN. 2014. A repeated-measures analysis of the effects of soft tissues on wrist range of motion in the extant phylogenetic bracket of dinosaurs: implications for the functional origins of an automatic wrist folding mechanism in crocodilia. Anat Rec 297:1228–49. 10.1002/ar.22903 24664936

[bib22] Ibrahim N , Maganuco S, Sasso CD, Fabbri M, Auditore M, Bindellini G, Martill DM, Zouhri S, Mattarelli DA, Unwin DM. 2020. Tail-propelled aquatic locomotion in a theropod dinosaur. Nature 581:67–70. 32376955 10.1038/s41586-020-2190-3

[bib23] Inoue M , Mizuno T, Sakakibara T, Kato T, Yoshikawa T, Inaba T, Kasai Y. 2017. Trajectory of instantaneous axis of rotation in fixed lumbar spine with instrumentation. J Orthop Surg Res 12:1–8. 29145877 10.1186/s13018-017-0677-x PMC5689179

[bib24] Jones KE , Dickson BV, Angielczyk KD, Pierce SE. 2021. Adaptive landscapes challenge the “lateral-to-sagittal” paradigm for mammalian vertebral evolution. Curr Biol 31:1883–92.e7. 33657406 10.1016/j.cub.2021.02.009

[bib25] Jones KE , Gonzalez S, Angielczyk KD, Pierce SE. 2020. Regionalization of the axial skeleton predates functional adaptation in the forerunners of mammals. Nat Ecol Evol 4:470–8. 32015524 10.1038/s41559-020-1094-9

[bib26] Jurestovsky DJ , Jayne BC, Astley HC. 2020. Experimental modification of morphology reveals the effects of the zygosphene–zygantrum joint on the range of motion of snake vertebrae. J Exp Biol 223:jeb216531. 32127376 10.1242/jeb.216531

[bib27] Kambic RE , Biewener AA, Pierce SE. 2017. Experimental determination of three-dimensional cervical joint mobility in the avian neck. Front Zool 14:1–15. 28747987 10.1186/s12983-017-0223-z PMC5525307

[bib28] Kambic RE , Roberts TJ, Gatesy SM. 2017. 3-D range of motion envelopes reveal interacting degrees of freedom in avian hind limb joints. J Anat 231:906–20. 28833095 10.1111/joa.12680 PMC5696129

[bib29] Kingma I , Busscher I, van der Veen AJ, Verkerke GJ, Veldhuizen AG, Homminga J, van Dieën JH. 2018. Coupled motions in human and porcine thoracic and lumbar spines. J Biomech 70:51–8. 29246473 10.1016/j.jbiomech.2017.11.034

[bib30] Knudson D . 2007. Fundamentals of biomechanics. New York, NY: Springer Science & Business Media.

[bib31] Koob TJ , Long JH. 2000. The vertebrate body axis: evolution and mechanical function. Am Zool 40:1–18.

[bib32] Kuznetsov AN , Tereschenko VS. 2010. A method for estimation of lateral and vertical mobility of platycoelous vertebrae of tetrapods. Paleontolog J 44:209–25.

[bib33] Lai PH , Biewener AA, Pierce SE. 2018. Three-dimensional mobility and muscle attachments in the pectoral limb of the Triassic cynodont *Massetognathus pascuali* (Romer, 1967). J Anat 232:383–406. 29392730 10.1111/joa.12766 PMC5807948

[bib34] Lauder GV , Thomason JJ. 1995. On the inference of function from structure. In: Functional morphology in vertebrate paleontology. Cambridge: Cambridge University Press. p. 1–18.

[bib35] Legaspi O , Edmond SL. 2007. Does the evidence support the existence of lumbar spine coupled motion? A critical review of the literature. J Orthop Sports Phys Ther 37:169–78. 17469669 10.2519/jospt.2007.2300

[bib36] Liebsch C , Wilke H-J. 2018. Basic biomechanics of the thoracic spine and rib cage.’ In: Biomechanics of the spine. Cambridge, MA: Academic Press.

[bib37] Long JH , Pabst DA, Shepherd WR, McLellan WA. 1997. Locomotor design of dolphin vertebral columns: bending mechanics and morphology of *Delphinus delphis*. J Exp Biol 200:65–81. 9023994 10.1242/jeb.200.1.65

[bib38] Lungmus JK , Angielczyk KD. 2019. Antiquity of forelimb ecomorphological diversity in the mammalian stem lineage (Synapsida). Proc Natl Acad Sci 116:6903–7. 30886085 10.1073/pnas.1802543116 PMC6452662

[bib39] Mallison H . 2010a. ‘CAD assessment of the posture and range of motion of *Kentrosaurus aethiopicus* Hennig 1915. Swiss J Geosci 103:211–33.

[bib40] Mallison H . 2010b. The digital Plateosaurus II: an assessment of the range of motion of the limbs and vertebral column and of previous reconstructions using a digital skeletal mount. Acta Palaeontol Pol 55:433–58.

[bib41] Manafzadeh AR , Kambic RE, Gatesy SM. 2021. A new role for joint mobility in reconstructing vertebrate locomotor evolution. Proc Natl Acad Sci 118:e2023513118. 33558244 10.1073/pnas.2023513118 PMC7896293

[bib42] McHenry CR , Wroe S, Clausen PD, Moreno K, Cunningham E. 2007. Supermodeled sabercat, predatory behavior in Smilodon fatalis revealed by high-resolution 3D computer simulation. Proc Natl Acad Sci 104:16010–5. 17911253 10.1073/pnas.0706086104 PMC2042153

[bib43] Molnar JL , Pierce SE, Hutchinson JR. 2014. An experimental and morphometric test of the relationship between vertebral morphology and joint stiffness in Nile crocodiles (*Crocodylus niloticus*). J Exp Biol 217:758–68. 24574389 10.1242/jeb.089904

[bib44] Molnar JL , Hutchinson JR, Diogo R, Clack JA, Pierce SE. 2021. Evolution of forelimb musculoskeletal function across the fish-to-tetrapod transition. Sci Adv 7:eabd7457. 33523947 10.1126/sciadv.abd7457 PMC10964964

[bib45] Molnar JL , Pierce SE, Bhullar B-AS, Turner AH, Hutchinson JR. 2015. Morphological and functional changes in the vertebral column with increasing aquatic adaptation in crocodylomorphs. R Soc Open Sci 2:150439. 26716001 10.1098/rsos.150439 PMC4680616

[bib46] Muñoz MM , Price SA. 2019. The future is bright for evolutionary morphology and biomechanics in the era of big data. Integr Comp Biol 59:599–603. 31353403 10.1093/icb/icz121

[bib47] Nagesan RS , Henderson DM, Anderson JS. 2018. A method for deducing neck mobility in plesiosaurs, using the exceptionally preserved *Nichollssaura borealis*. R Soc Open Sci 5:172307. 30224996 10.1098/rsos.172307 PMC6124041

[bib48] Nelson G , Ellis S. 2019. The history and impact of digitization and digital data mobilization on biodiversity research. Philos Trans R Soc B, 374:20170391. 10.1098/rstb.2017.0391 PMC6282090 30455209

[bib49] Nowroozi BN , Harper CJ, De Kegel B, Adriaens D, Brainerd EL. 2012. Regional variation in morphology of vertebral centra and intervertebral joints in striped bass, *Morone saxatilis*. J Morphol 273:441–52. 22109664 10.1002/jmor.11034

[bib50] Nyakatura JA , Melo K, Horvat T, Karakasiliotis K, Allen VR, Andikfar A, Andrada E, Arnold P, Lauströer J, Hutchinson JR. 2019. Reverse-engineering the locomotion of a stem amniote. Nature 565:351. 30651613 10.1038/s41586-018-0851-2

[bib51] Oliver JD , Jones KE, Hautier L, Loughry WJ, Pierce SE. 2016. Vertebral bending mechanics and xenarthrous morphology in the nine-banded armadillo (*Dasypus novemcinctus*). J Exp Biol 219:2991–3002. 27473436 10.1242/jeb.142331

[bib52] Penning L , Badoux DM. 1987. Radiological study of the movements of the cervical spine in the dog compared with those in man. Anat Histol Embryol 16:1–20. 2954487 10.1111/j.1439-0264.1987.tb00720.x

[bib53] Pierce SE , Clack JA, Hutchinson JR. 2011. Comparative axial morphology in pinnipeds and its correlation with aquatic locomotory behaviour. J Anat 219:502–14. 21668895 10.1111/j.1469-7580.2011.01406.x PMC3196755

[bib54] Pierce SE , Clack JA, Hutchinson JR. 2012. Three-dimensional limb joint mobility in the early tetrapod Ichthyostega. Nature 486:523–6. 22722854 10.1038/nature11124

[bib55] R Development Core Team . 2009. R: a language and environment for statistical computing.

[bib56] Reilly SM , Elias JA. 1998. Locomotion in *Alligator mississippiensis*: kinematic effects of speed and posture and their relevance to the sprawling-to-erect paradigm. J Exp Biol 201:2559–74. 9716509 10.1242/jeb.201.18.2559

[bib57] Samagh SP , Rosen CD, Otarodifard K, Kornswiet M, Palmer G, Lee TQ. 2011. New method for determining apparent axial center of rotation of lumbar and thoracic spine segments. J Rehabil Res Dev 48:587–96. 21674408 10.1682/jrrd.2010.09.0168

[bib58] Schilling N . 2011. Evolution of the axial system in craniates: morphology and function of the perivertebral musculature. Front Zool 8:4–23. 21306656 10.1186/1742-9994-8-4 PMC3041741

[bib59] Schilling N , Hackert R. 2006. Sagittal spine movements of small therian mammals during asymmetrical gaits. J Exp Biol 209:3925–39. 16985208 10.1242/jeb.02400

[bib60] Schmidt H , Heuer F, Claes L, Wilke H-J. 2008. The relation between the instantaneous center of rotation and facet joint forces: a finite element analysis. Clin Biomech (Bristol, Avon) 23:270–8. 10.1016/j.clinbiomech.2007.10.001 17997207

[bib61] Selbie WS , Thomson DB, Richmond FJR. 1993. Sagittal-plane mobility of the cat cervical spine. J Biomech 26:917–27. 8349717 10.1016/0021-9290(93)90054-i

[bib62] Sellers WI , Manning PL, Lyson T, Stevens K, Margetts L. 2009. Virtual palaeontology: gait reconstruction of extinct vertebrates using high performance computing. Palaeontol Electron 12:12.3.

[bib63] Slijper EJ . 1946. Comparative biologic-anatomical investigations on the vertebral column and spinal musculature of mammals. Amsterdam: North-Holland Pub. Co. p. 1–128.

[bib64] Stevens KA , Parrish JM. 1999. Neck posture and feeding habits of two Jurassic sauropod dinosaurs. Science 284:798–800. 10221910 10.1126/science.284.5415.798

[bib65] Stevens KA , Parrish JM. 2005. Digital reconstructions of sauropod: dinosaurs and implications for feeding.’ In: The sauropods: evolution and paleobiology. Berkeley (CA): University of California Press.

[bib66] Taylor MP , Wedel MJ. 2013. The effect of intervertebral cartilage on neutral posture and range of motion in the necks of sauropod dinosaurs. PLoS One 8:e78214. 24205163 10.1371/journal.pone.0078214 PMC3812996

[bib67] Taylor MP , Wedel MJ, Naish D. 2009. Head and neck posture in sauropod dinosaurs inferred from extant animals. Acta Palaeontol Pol 54:213–20.

[bib68] Tsai HP , Turner ML, Manafzadeh AR, Gatesy SM. 2020. Contrast-enhanced XROMM reveals *in vivo* soft tissue interactions in the hip of Alligator mississippiensis. J Anat 236:288–304. 31691966 10.1111/joa.13101 PMC6956439

[bib69] Vidal D , Mocho P, Páramo A, Sanz JL, Ortega F. 2020. Ontogenetic similarities between giraffe and sauropod neck osteological mobility. PLoS One 15:e0227537. 31929581 10.1371/journal.pone.0227537 PMC6957182

[bib70] Wachs K , Fischer MS, Schilling N. 2016. Three-dimensional movements of the pelvis and the lumbar intervertebral joints in walking and trotting dogs. Vet J 210:46–55. 26831181 10.1016/j.tvjl.2015.12.009

[bib71] Werneburg I , Hinz JK, Gumpenberger M, Volpato V, Natchev N, Joyce WG. 2015. Modeling neck mobility in fossil turtles. J Exp Zool B Mol Dev Evol 324:230–43. 24497449 10.1002/jez.b.22557

[bib72] White III AA , Panjabi MM. 1990. Clinical biomechanics of the spine. Philadelphia (PA): Lippincott Williams & Wilkins.

[bib73] Willey JS , Biknevicius AR, Reilly SM, Earls KD. 2004. The tale of the tail: limb function and locomotor mechanics in Alligator mississippiensis. J Exp Biol 207:553–63. 14691103 10.1242/jeb.00774

[bib74] Wintrich T , Jonas R, Wilke H-J, Schmitz L, Sander PM. 2019. Neck mobility in the Jurassic plesiosaur *Cryptoclidus eurymerus*: finite element analysis as a new approach to understanding the cervical skeleton in fossil vertebrates. PeerJ 7:e7658. 31720095 10.7717/peerj.7658 PMC6842296

[bib75] Wintrich T , Scaal M, Böhmer C, Schellhorn R, Kogan I, van der Reest A, Sander PM. 2020. Palaeontological evidence reveals convergent evolution of intervertebral joint types in amniotes. Sci Rep 10:1–14. 32839497 10.1038/s41598-020-70751-2 PMC7445751

[bib76] Xia Q , Wang S, Kozanek M, Passias P, Wood K, Li G. 2010. *In vivo* motion characteristics of lumbar vertebrae in sagittal and transverse planes. J Biomech 43:1905–9. 20381051 10.1016/j.jbiomech.2010.03.023

