# Supplementary material for: AutoBend: An Automated Approach for Estimating Intervertebral Joint Function from Bone-Only Digital Models
Source: Integr Org Biol. 2021 Oct 13;3(1):obab026. doi: 10.1093/iob/obab026 (PMC8514422; doi:10.1093/iob/obab026)
Supplement: obab026_Supplemental_Files [file obab026_supplemental_files.zip › Abstract_Spanish and Portugese.docx]

**Abstract (400 words)**

Deciphering the biological function of rare or extinct species is key to understanding evolutionary patterns across the tree of life. While soft tissues are vital determinants of joint function, they are rarely available for study. Therefore, extracting functional signals from skeletons, which are more widely available via museum collections, has become a priority for the field of comparative biomechanics. While most work has focused on the limb skeleton, the axial skeleton plays a critical role in body support, respiration, and locomotion, and is therefore of central importance for understanding broad-scale functional evolution. Here, we describe and experimentally validate *AutoBend,*an automated approach to estimating intervertebral joint function from bony vertebral columns. *AutoBend* calculates osteological range of motion (oROM) by automatically manipulating digitally articulated vertebrae while incorporating multiple constraints on motion, including both bony intersection and the role of soft tissues by restricting excessive strain in both centrum and zygapophyseal articulations. Using *AutoBend* and biomechanical data from cadaveric experiments on cats and tegus, we validate important modeling parameters required for oROM estimation, including the degree of zygapophyseal disarticulation, and the location of the center of rotation. Based on our validation, we apply a model with the center of rotation located within the vertebral disc, no joint translation, around 50% strain permitted in both zygapophyses and discs, and a small amount of vertebral intersection permitted. Our approach successfully reconstructs magnitudes and craniocaudal patterns of motion obtained from *ex vivo* experiments, supporting its potential utility. It also performs better than more typical methods that rely solely on bony intersection, emphasizing the importance of accounting for soft tissues. We estimated the sensitivity of the analyses to vertebral model reconstruction by varying joint spacing, degree of overlap, and the impact of landmark placement. The effect of these factors was small relative to biological variation craniocaudally and between bending directions. Within, we also present a new approach for estimating joint stiffness directly from oROM and morphometric measurements that can successfully reconstruct the craniocaudal patterns, but not magnitudes, derived from experimental data. Together, this work represents a significant step forward for understanding vertebral function in difficult-to-study (e.g., rare or extinct) species, paving the way for a broader understanding of patterns of functional evolution in the axial skeleton.

**Resumo [Portuguese]**

Decifrar a função biológica de espécies raras ou extintas é fundamental para se compreender os padrões evolutivos na árvore da vida. Embora os tecidos moles sejam determinantes vitais das funções articulares, estes raramente estão disponíveis para estudo. Portanto, extrair dados funcionais provenientes de esqueletos, que são mais amplamente disponíveis por meio de coleções de museus, tornou-se uma prioridade para o campo da biomecânica comparada. Embora a maioria dos trabalhos biomecânicos tenham focado no esqueleto apendicular, o esqueleto axial também desempenha um papel crítico para o suporte corporal, respiração e locomoção e, portanto, é de importância central para a compreensão da evolução funcional em escalas amplas. Nesse trabalho, nós descrevemos e validamos experimentalmente o *AutoBend*, uma abordagem automatizada para estimar a função da articulação intervertebral de colunas vertebrais ósseas. O *AutoBend* calcula a amplitude do movimento osteológico (AMO) manipulando automaticamente as vértebras reconstruídas digitalmente e incorporando várias restrições de movimento, incluindo restrições das interseções ósseas e o papel de restrição dos tecidos moles na tensão excessiva sobre as articulações dos centros vertebrais e zigapofisárias. Usando *AutoBend* e dados biomecânicos de experimentos cadavéricos em gatos e lagartos tegus, validamos parâmetros de modelagem importantes e necessários para as estimativa do AMO, incluindo o grau de desarticulação zigapofisária e a localização do centro de rotação. Com base nessa validação, aplicamos um modelo com o centro de rotação localizado dentro do disco vertebral, sem translação articular, com cerca de 50% de tensão permitida nas zigapófises e discos vertebrais, além de uma pequena quantidade de intersecção vertebral permitida. Nossa abordagem reconstrói com sucesso magnitudes e padrões de movimento craniocaudais obtidos a partir de experimentos *ex vivo*, corroborando a sua potencial utilidade. Esse modelo também tem um desempenho melhor do que os métodos mais típicos que dependem apenas das interseções ósseas, enfatizando a importância de se levar em conta o papel dos tecidos moles. Estimamos a sensibilidade das análises à reconstrução do modelo vertebral, variando o espaçamento entre articulações, o grau de sobreposição e o impacto da localização dos pontos de referência. O efeito desses fatores foi pequeno em relação à variação biológica craniocaudal e entre as direções de flexão. Apresentamos aqui também uma nova abordagem para se estimar a rigidez articular diretamente à partir do AMO e medidas morfométricas que podem reconstruir com sucesso os padrões craniocaudais (embora não as magnitudes) derivados de dados experimentais. Este trabalho representa um passo significativo para a melhor compreensão da função vertebral em espécies difíceis de estudar (por exemplo, raras ou extintas), abrindo caminho para uma compreensão mais ampla dos padrões de evolução funcional no esqueleto axial.

**Resumen [Spanish]**

Descifrar la función biológica de especies raras o extintas es fundamental para comprender los patrones evolutivos del árbol de la vida. Aunque los tejidos blandos son determinantes vitales de la función articular, raramente están disponibles para su estudio. Por lo tanto, la extracción de datos funcionales de esqueletos, que están más comumente disponibles a través de colecciones de museos, se ha convertido en una prioridad para el campo de la biomecánica comparada. Aunque la mayor parte del trabajo biomecánico se ha centrado en el esqueleto apendicular, el esqueleto axial también desempeña un papel fundamental para el soporte del cuerpo, la respiración y la locomoción y, por lo tanto, es de vital importancia para comprender la evolución funcional a gran escala. En este trabajo, describimos y validamos experimentalmente *AutoBend*, una herramienta automatizada para estimar la función de la articulación intervertebral en las columnas vertebrales óseas. *AutoBend* calcula el rango de movimiento osteológico (RMOo) manipulando automáticamente las vértebras reconstruidas digitalmente e incorporando varias restricciones de movimiento, incluidas las restricciones de intersección ósea y el papel de la restricción de tejidos blandos en la tensión excesiva sobre las articulaciones cigapofisarias y centros vertebrales. Utilizando *AutoBend* y datos biomecánicos de experimentos cadavéricos en gatos y lagartos tegus, validamos importantes parámetros de modelado necesarios para estimar el RMOo, incluido el grado de desarticulación cigapofisaria y la ubicación del centro de rotación. Con base en esta validación, aplicamos un modelo con el centro de rotación ubicado dentro del disco vertebral, sin traslación articular, con 50% de tensión permisible en la cigapófisis y los discos vertebrales, además de una pequeña cantidad de intersección vertebral permitida. Nuestra herramienta reconstruye con éxito magnitudes craneocaudales y patrones de movimiento obtenidos de experimentos *ex vivo*, corroborando su potencial utilidad. Este modelo también funciona mejor que los métodos más típicos que se basan solo en las intersecciones óseas, enfatizando la importancia de tener en cuenta el papel de los tejidos blandos. Estimamos la sensibilidad de los análisis a la reconstrucción del modelo vertebral, variando el espaciamiento entre articulaciones, el grado de superposición y el impacto de la ubicación de los puntos de referencia. El efecto de estos factores fue pequeño en relación a la variación biológica craneocaudal y entre las direcciones de flexión. También presentamos aquí un nuevo enfoque para estimar la rigidez articular directamente de la RMOo y mediciones morfométricas que pueden reconstruir con éxito los patrones craneocaudales (aunque no las magnitudes) derivados de datos experimentales. Este trabajo representa un paso significativo hacia una mejor comprensión de la función vertebral en especies difíciles de estudiar (por ejemplo, raras o extintas), allanando el camino para una comprensión más amplia de los patrones de evolución funcional en el esqueleto axial.
