# Supplementary material for: AutoBend: An Automated Approach for Estimating Intervertebral Joint Function from Bone-Only Digital Models
Source: Integr Org Biol. 2021 Oct 13;3(1):obab026. doi: 10.1093/iob/obab026 (PMC8514422; doi:10.1093/iob/obab026)
Supplement: obab026_Supplemental_Files [file obab026_supplemental_files.zip › Supplemental_6.8.2021.docx]

***Table S1. ANOVA table.*** *Analysis of variance examining the impact of model parameters (Joint spacing, intersection threshold, strain, translation) relative to the main effects of joint, direction, and their interaction (Joint:Direction). Though significant, the effect size of model parameters is small relative to the main effects (Figure 8).*

|  | *Felis catus* | | | | | *Salvator merianae* | | | | |
| --- | --- | --- | --- | --- | --- | --- | --- | --- | --- | --- |
|  | **Df** | **Sum Sq** | **Mean Sq** | **F value** | **P value** | **Df** | **Sum Sq** | **Mean Sq** | **F value** | **P value** |
| **Joint** | 24 | 10326.8 | 430.3 | 158.0 | >0.001 | 22 | 2722.4 | 123.7 | 46.6 | >0.001 |
| **Direction** | 2 | 3510.0 | 1755.0 | 644.3 | >0.001 | 2 | 55199.5 | 27599.7 | 10389.2 | >0.001 |
| **Joint Spacing** | 1 | 169.6 | 169.6 | 62.3 | >0.001 | 1 | 104.8 | 104.8 | 39.4 | >0.001 |
| **Int. Thresh** | 1 | 436.9 | 436.9 | 160.4 | >0.001 | 1 | 162.5 | 162.5 | 61.2 | >0.001 |
| **Strain** | 1 | 418.3 | 418.3 | 153.6 | >0.001 | 1 | 366.0 | 366.0 | 137.8 | >0.001 |
| **Joint:Direction** | 48 | 43042.4 | 896.7 | 329.2 | >0.001 | 44 | 3534.9 | 80.3 | 30.2 | >0.001 |
| **Residuals** | 1121 | 1421.8 | 2.7 | - | - | 480 | 1275.2 | 2.7 | - | - |


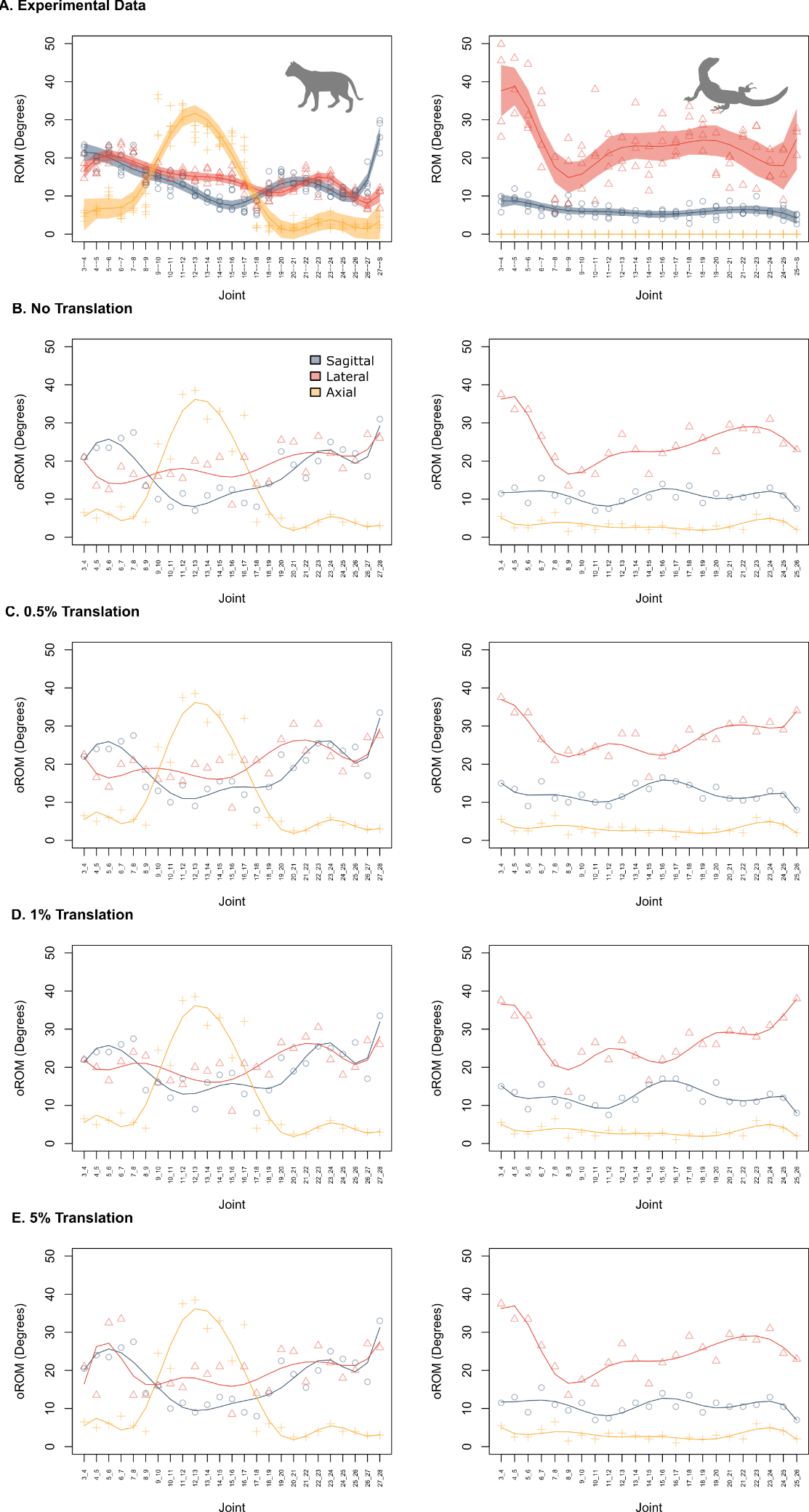


**Figure S1. Impact of translation on along column patterns of mobility. (A)** Experimental data. Shaded region: 95% confidence interval. **(B)** *AutoBend* with no translation. *AutoBend* with **(C)** 0.5%, **(D)** 1%, and **(E)** 5% translation by vertebral area.


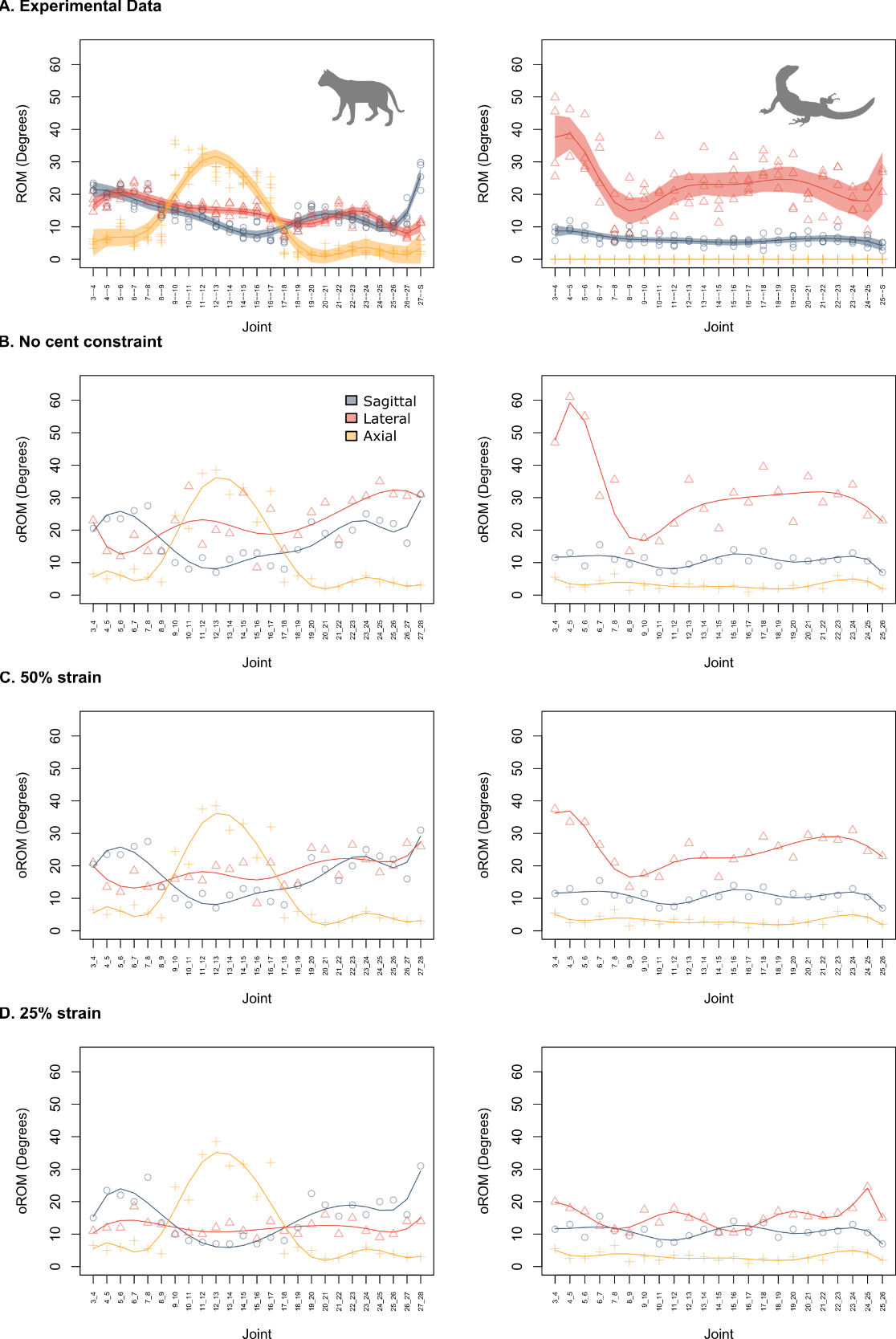


**Figure S2. Impact of centrum strain on along column patterns of mobility. (A)** Experimental data. Shaded region: 95% confidence interval. **(B)** *AutoBend* with no centrum constraints. *AutoBend* with **(C)**50% and **(D)** 25% strain permitted at the centrum.


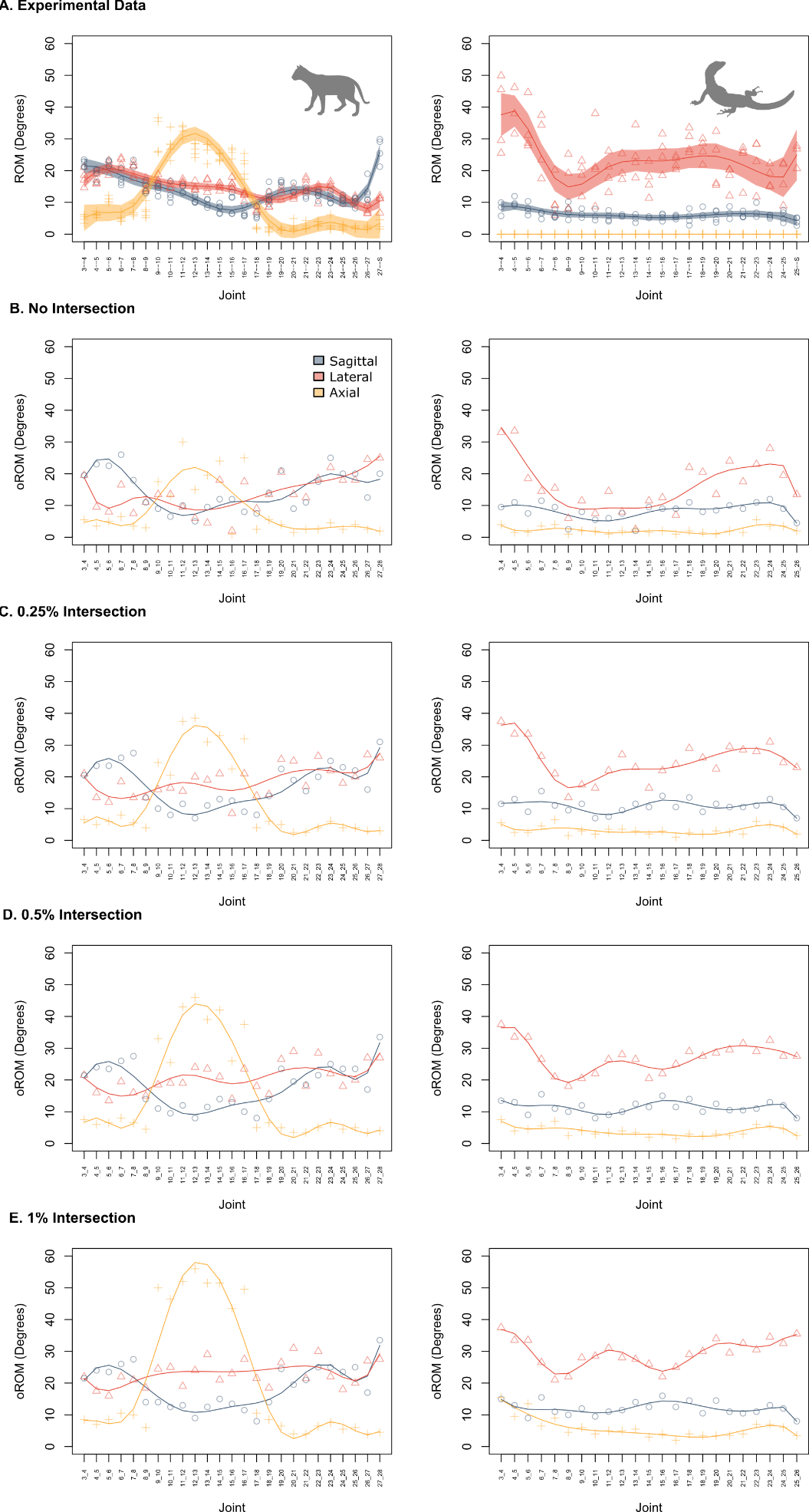


**Figure S3. Impact of intersection threshold on along column patterns of mobility. (A)** Experimental data. Shaded region: 95% confidence interval. **(B)** *AutoBend* with no bony intersection permitted. *AutoBend* with **(C)** 0.25%, **(D)** 0.5%, and **(E)** 1% intersection threshold based on vertebral area.
